# Mutual exclusivity of *ESR1* and *TP53* mutations in endocrine resistant metastatic breast cancer

**DOI:** 10.1038/s41523-022-00426-w

**Published:** 2022-05-10

**Authors:** Zheqi Li, Nicole S. Spoelstra, Matthew J. Sikora, Sharon B. Sams, Anthony Elias, Jennifer K. Richer, Adrian V. Lee, Steffi Oesterreich

**Affiliations:** 1 Department of Pharmacology and Chemical Biology, University of Pittsburgh, Pittsburgh, PA USA; 2 Women’s Cancer Research Center, Magee Women’s Research Institute, UPMC Hillman Cancer Center, Pittsburgh, PA USA; 3 Department of Pathology, University of Colorado Anschutz Medical Campus, Aurora, CO USA; 4 School of Medicine, Division of Oncology, University of Colorado Anschutz Medical Campus, Aurora, CO USA

**Keywords:** Breast cancer, Epistasis, Transcription

## Abstract

Both *TP53* and *ESR1* mutations occur frequently in estrogen receptor positive (ER+) metastatic breast cancers (MBC) and their distinct roles in breast cancer tumorigenesis and progression are well appreciated. Recent clinical studies discovered mutual exclusivity between *TP53* and *ESR1* mutations in metastatic breast cancers; however, mechanisms underlying this intriguing clinical observation remain largely understudied and unknown. Here, we explored the interplay between *TP53* and *ESR1* mutations using publicly available clinical and experimental data sets. We first confirmed the robust mutational exclusivity using six independent cohorts with 1,056 ER+ MBC samples and found that the exclusivity broadly applies to all ER+ breast tumors regardless of their clinical and distinct mutational features. *ESR1* mutant tumors do not exhibit differential p53 pathway activity, whereas we identified attenuated ER activity and expression in *TP53* mutant tumors, driven by a p53-associated E2 response gene signature. Further, 81% of these p53-associated E2 response genes are either direct targets of wild-type (WT) p53-regulated transactivation or are mutant p53-associated microRNAs, representing bimodal mechanisms of ER suppression. Lastly, we analyzed the very rare cases with co-occurrences of *TP53* and *ESR1* mutations and found that their simultaneous presence was also associated with reduced ER activity. In addition, tumors with dual mutations showed higher levels of total and PD-L1 positive macrophages. In summary, our study utilized multiple publicly available sources to explore the mechanism underlying the mutual exclusivity between *ESR1* and *TP53* mutations, providing further insights and testable hypotheses of the molecular interplay between these two pivotal genes in ER+ MBC.

## Introduction

Breast cancer is the leading cause of cancer-related death in women worldwide^1^ and ER positive (ER+) breast cancer accounts for approximately two-thirds of all cases^2,3^. Endocrine treatment is the current mainstay of therapy for patients with ER+ breast cancers^3,4^. Despite decades-long benefit of endocrine therapy, the development of endocrine resistance in part due to the complex nature of cancer heterogeneity remains a large clinical and social-economic issue^4,5^.

It is well-established that cancer is initiated and promoted by the accumulation of genetic mutations under selection of the ecosystem^6–8^. A founder mutation typically undergoes clonal expansion to engender tumorigenesis, whereas continuous generation of passenger mutations results in diverse tumor-favorable phenotypes during evolution to overcome environmental burdens such as therapeutic pressure and clonal competition^9–11^. In the context of ER+ breast cancer, mutations in *TP53* occur in approximately 30% cases and are widely considered as one of the most essential drivers of tumor initiation^12,13^. Inactivation of p53 is known to result in multiple cellular consequences including cell cycle promotion, abrogation of apoptosis, and DNA repair disruption, which may ultimately accelerate tumor progression and therapeutic resistance^14^. Besides the suppressive role on canonical p53 function, a subclass of *TP53* mutation variants exhibit gain-of-function (GoF). These GoF mutations render additional features to cancer cells such as enhanced invasiveness and metabolic reprogramming to facilitate tumor progression^15–17^. Unlike *TP53* mutations which have been investigated for decades, only recent studies have provided in-depth characterization of hotspot mutations in *ESR1*, the gene encoding estrogen receptor-α^18–20^. *ESR1* mutations rarely occur in primary tumors but are strongly enriched in approximately 30–40% of endocrine-resistant MBC^20–22^. Pre-clinical investigations by our groups and other have shown that these mutations cause not only ligand-independent ER activation but also phenotypical advantages that lead to metastatic progression in the face of endocrine therapy^23–25^.

Breast cancer is a disease with an extensive degree of genetic heterogeneity, and the epistatic relationship between two mutations may drive inter- and intra-clonal cooperation and competition^26^. Two co-occurring mutations typically imply collaborative interaction of two oncogenic pathways, such as *MYC* and *TP53* mutations in breast cancer^27^, and *BRAF* and *PTEN* mutations in melanoma^28^. In contrast, two mutations showing mutual exclusivity may represent either functional redundancy or antagonism^29^. The former is exemplified by the recently reported exclusivity between mutations of *ESR1* and multiple MAPK pathway genes in ER+ metastatic cacers^30^, while the exclusivity of *PTEN* loss and *CHD1* mutations in breast and prostate cancer reflects functional antagonism^31^. Importantly, both mutational co-occurrence and mutual exclusivity may designate potential therapeutic vulnerability. First, the joint targeting of two co-occurring driver mutant genes and their associated pathways is a long-standing endeavor in clinic. Investigation of mutually exclusive gene mutations may reveal unique synthetic lethal dependency of a certain driver gene, and therapies towards activation of the counterpart may be exploited. For example, identification of genes synthetically lethal with loss of *CDH1* in breast cancer has led to preclinical validation and clinical trials^32^.

Two recent studies have reported mutual exclusivity between *ESR1* and *TP53* mutations in MBC^23,33^; however, the mechanisms underlying this observation are poorly understood. Molecular interactions between ER and p53 in breast cancer have been previously characterized as a bi-modal loop. Studies using pre-clinical models reported that ER directly binds to p53 and subsequently blocks its transactivation by recruiting corepressors and histone deacetylase^34,35^. Further studies showed that estradiol could prevent p53-mediated apoptosis^36^. Conversely, WT p53 was also reported to be involved in *ESR1* transactivation through binding to promoter regions^37,38^.

In this study, we utilized recently generated genomic data from MBC clinical samples to better understand the mutual exclusivity between *ESR1* and *TP53* mutations. We observed an epistatic relationship between *ESR1* and *TP53* mutations in six independent cohorts and further identified a unidirectional inhibitory effect of mutant p53 on ER signaling via either loss of transcriptional activation or microRNA-mediated repression. These studies provide hypothesis-generating data on the mechanistic interplay between *ESR1* and *TP53* mutations in ER+ MBC that may be of clinical relevance.

## Results

### *ESR1* and *TP53* mutations are mutually exclusive in MBC

**Fig. 1:**
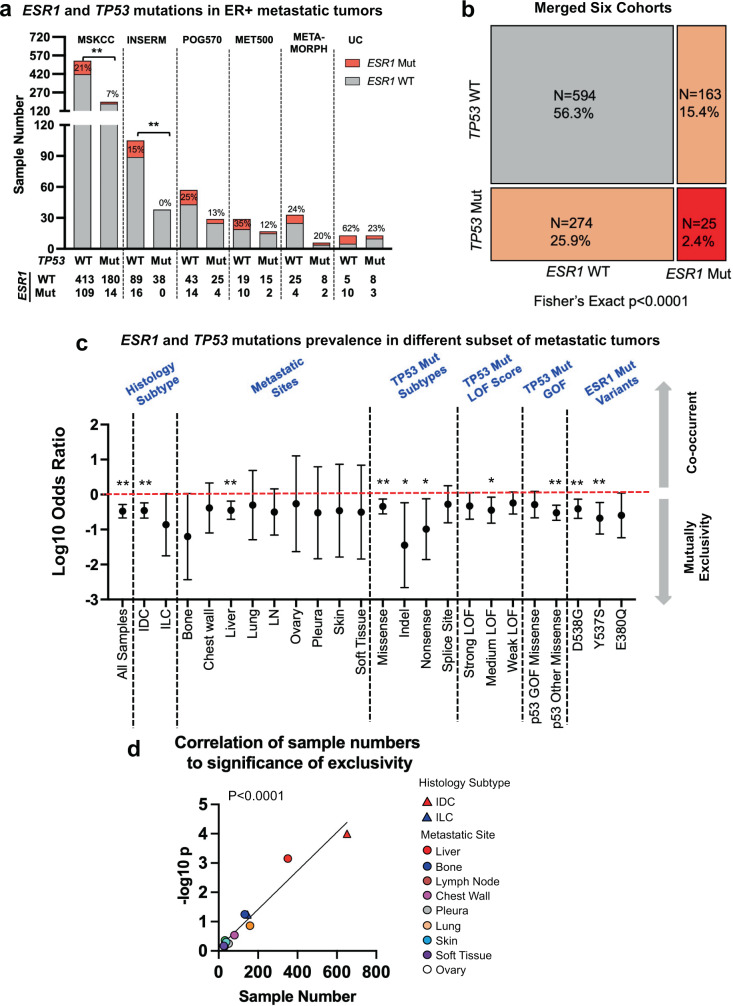
*ESR1* and *TP53* mutations are mutually exclusive in metastatic breast cancer. **a** Stacked bar plot representing numbers of *ESR1* mutant tumors cross with *TP53* WT and mutant subsets among six independent cohorts. Only ER+ metastatic samples were selected for this analysis. Specific numbers of each portion were labeled below. Fisher’s exact test was performed towards each cohort. (**p < 0.01). **b** Mosaic plot showing the association between *ESR1* and *TP53* genotype status merged from all six cohorts. Fisher’s exact test was applied. **c** Forest plot representing the odds ratio of *ESR1* and *TP53* mutations within each specific subset of comparison. Error bars represent 95% CI. Each comparison utilized the merged data set of all six cohorts indicated above. Fisher’s exact test (two-sided) was used. (*p < 0.05; **p < 0.01). **d** Dot plot showing the correlation of log10 *p* values of Fisher’s exact test from each subset analysis to the sample size of each subset. Pearson correlation analysis was performed for all the data points.

Two previous studies reported mutual exclusivity of *ESR1* and *TP53* mutations in MBC^23,33^. To examine the robustness of this clinical observation, we expanded the analysis to six previously reported MBC cohorts (*n* = 1056 ER+ MBC cases)^23,30,39–42^ (Fig. 1a). We observed significant (*p* < 0.0001, Fishers exact test) mutual exclusivity between *ESR1* and *TP53* mutations (Fig. 1b) with co-occurrence of mutation in only 25 breast cancers out of 1056 examined.

To further determine whether the intriguing exclusivity is restricted to breast cancers with particular clinical or genetic features, we reassessed the prevalence of *ESR1* and *TP53* mutations in different subsets of patients (Supplementary Table 1). First, *ESR1* and *TP53* mutations were mutually exclusive regardless of their histological subtypes or distant metastatic sites (Fig. 1c). Notably, the statistical significance was tightly correlated with the size of these subsets (Fig. 1d). Furthermore, the mutual exclusivity was not related to the type of *TP53* mutation, the extent of loss-of-function, gain of function status^43^, nor the type of *ESR1* mutation (Fig. 1c). However, distribution analysis under the same setting revealed that tumors harboring both mutations are more associated with liver metastasis and *TP53* missense and splice site mutations (Supplementary Fig. 1). Taken together, in this multi-cohort analysis, we confirmed the strong and robust mutual exclusivity between *ESR1* and *TP53* mutations in MBC, and this phenomenon is broadly observed in all ER+ MBC tumor types, while the rare occurrence of dual mutation-tumors is associated with unique clinical and genomic features.

### *ESR1* mutations do not affect p53 expression or downstream signaling pathways

**Fig. 2:**
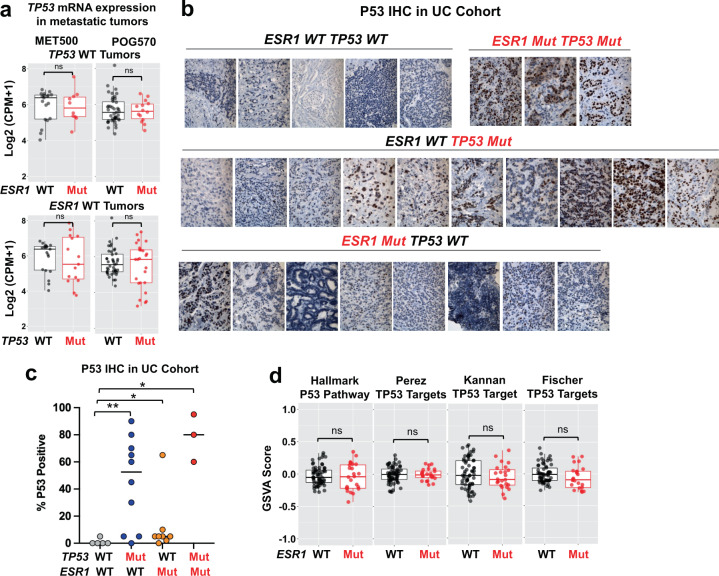
*ESR1* mutations do not affect *TP53* expression or *TP53* pathway activity in *TP53* WT ER + metastatic tumors. **a** Box plot showing *TP53* mRNA expression between *ESR1* WT (*n* = 19 for MET500; *n* = 43 for POG570) and mutant (*n* = 10 for MET500; *n* = 14 for POG570) in *TP53* WT tumors (upper panel) and *TP53* mRNA expression between *TP53* WT (*n* = 19 for MET500; *n* = 43 for POG570) and mutant tumor (*n* = 15 for MET500; *n* = 25 for POG570) in *ESR1* WT tumors (lower panel) in MET500 and POG570 cohorts. Log2(CPM + 1) values for *TP53* gene were extracted from RNA-seq. Box plots span the upper quartile (upper limit), median (center), and lower quartile (lower limit). Whiskers extend a maximum of 1.5× IQR. Mann Whitney U test (two-sided) was applied to each comparison. **b** Representative images of p53 immunohistochemistry staining of 26 ER+ metastatic tumors from UC cohort. Images were classified by the genotype of *ESR1* and *TP53*. **c** Dot plots representing p53 IHC quantifications of B in four different groups. Median of each group was indicated. Mann–Whitney U test (two-sided) was used (*p < 0.05; **p < 0.01). **d** Box plots representing the enrichment levels of four different p53-associated gene signatures between *TP53* WT; *ESR1* WT (*n* = 62) and *TP53* WT; *ESR1* mutant (*n* = 24) ER+ tumors from merged MET500 and POG570 cohort. Box plots span the upper quartile (upper limit), median (center), and lower quartile (lower limit). Whiskers extend a maximum of 1.5× IQR. Mann–Whitney U test (two-sided) was used.

Potential explanations for mutation mutual exclusivity are functional redundancy or antagonism^26^. We next sought to address the mechanism underpinning the clinical observation by examining the impact of *ESR1* mutations on downstream *TP53* signaling networks. We hypothesized that *ESR1* mutations in MBC alter p53 signaling activity and thus lead to their incompatibility and mutual exclusivity. We integrated transcriptomic profiles from two of the largest cohorts of combined DNA and RNA profiling in MBC, namely POG570 and MET500, and examined *TP53* mRNA levels and function of the p53 pathway^40,41^. First, *TP53* mRNA levels were not altered in *ESR1* mutant tumors (Fig. 2a). Due to the pivotal role of p53 protein stability in determining its functions^44,45^, we further examined p53 protein expression using immunohistochemistry on 26 ER+ metastatic tumors with annotated mutations from the University of Colorado (UC) cohort (Fig. 2b). Consistent with the previous literature^46^, *TP53* mutant tumors showed increased p53 protein (Fig. 2c). On the other hand, among all *TP53* WT tumors, *ESR1* mutant tumors did not show substantial changes of p53 protein levels (below 20% positive cells) compared to *ESR1* WT tumors, though one tumor exhibited exceptional elevation (Fig. 2c). We next used four previously reported *TP53* gene signatures to delineate downstream effects^47–50^. While we found a strong repression of p53 signaling activity in *TP53* mutant tumors in TCGA as a reference (Supplementary Fig. 2a), *ESR1* mutations did not change the p53 signatures (Fig. 2d). Overall, this analysis indicates that *ESR1* mutations do not impact p53 function in metastatic tumors. Furthermore, we did not identify mutual exclusivity between mutations of *ESR1* and other members of the p53 signaling axis including *MDM2*, *MDM4*, *CDKN2A,* and *CDKN2B* (Supplementary Fig. 2b), suggesting that *ESR1*-*TP53* mutational exclusivity is presumably independent of p53 pathway alterations. Hence p53 canonical activity is unlikely to be the cause of the exclusivity.

### *TP53* mutation is inversely correlated with expression of ER-α and a subset of downstream genes

**Fig. 3:**
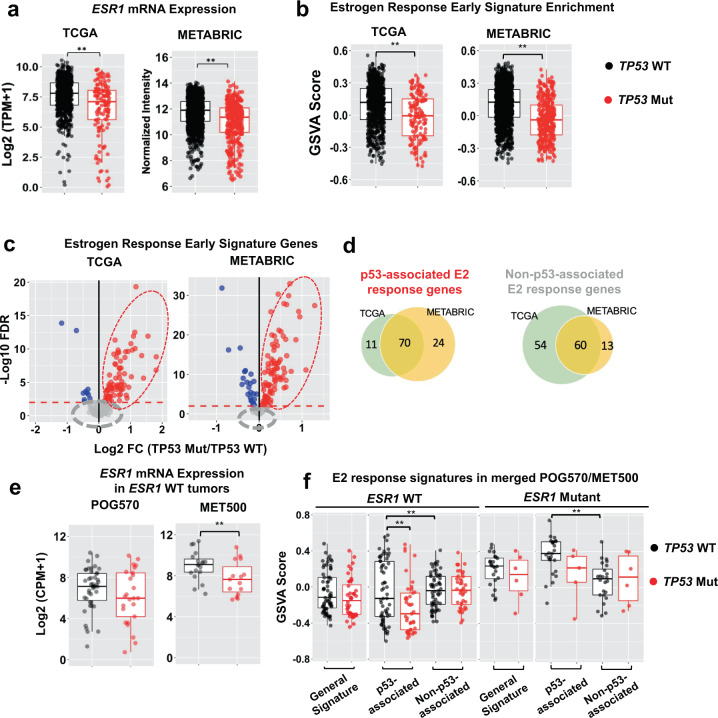
*TP53* mutation is inversely correlated with expression of ER-α and a subset of downstream genes. **a**, **b** Box plots representing the expression levels of *ESR1* gene (**a**) or enrichment levels of “Estrogen Response Early” signatures (**b**) in *TP53* WT versus *TP53* mutant ER+ primary tumors from TCGA (*n* = 672 *TP53* WT; *n* = 136 *TP53* Mut) and METABRIC (*n* = 1187 *TP53* WT; *n* = 318 *TP53* Mut) cohorts. Box plots span the upper quartile (upper limit), median (center), and lower quartile (lower limit). Whiskers extend a maximum of 1.5X IQR. Mann–Whitney U test (two-sided) was used (**p < 0.01). **c** Volcano plots showing differentially expressing genes within Estrogen Response Early signature (*n* = 200) in *TP53* mutant tumors versus WT tumors in TCGA and METABRIC breast cancer cohorts. DE genes were selected using the cutoff of FDR < 0.01. Genes that were upregulated, downregulated, or unchanged were labeled in red, blue, and gray respectively. **d** Venn diagram showing the overlap of mutant p53 positively associated (left panel) or unassociated (right panel) estrogen response genes between TCGA and METABRIC cohorts. The intersected 70 and 60 genes consist of the P53-ER Signature and Non-P53-ER Signature respectively. **e** Box plots representing the expression levels of *ESR1* gene in *TP53* WT (*n* = 19 for MET500; *n* = 43 for POG570) versus *TP53* mutant (*n* = 15 for MET500; *n* = 25 for POG570) metastatic tumors in MET500 and POG570 cohorts. Box plots span the upper quartile (upper limit), median (center), and lower quartile (lower limit). Whiskers extend a maximum of 1.5× IQR. Samples were pre-selected for *ESR1* WT genotype. Mann–Whitney U test (two-sided) was applied to each cohort (**p < 0.01). **f** Box plots representing the enrichment levels of general “Estrogen Response Early” signatures, P53-ER Signature, and Non-P53-ER Signature between *TP53* WT and mutant tumors in the separate contexts of *ESR1* WT (left panel, *n* = 62 *TP53* WT; *n* = 40 *TP53* Mut) and mutant (right panel, *n* = 24 *TP53* WT; *n* = 6 *TP53* Mut) tumors. GSVA scores were combined from MET500 and POG570 cohorts. Box plots span the upper quartile (upper limit), median (center), and lower quartile (lower limit). Whiskers extend a maximum of 1.5X IQR. Mann–Whitney U test (two-sided) was used for each comparison. (*p < 0.05; **p < 0.01).

We next explored the mechanism from an opposite regulatory direction. Since *TP53* mutations typically arise as founder mutations in primary tumors, we questioned whether pre-existing *TP53* mutant clones hinder the development of *ESR1* mutations by directly inhibiting ER signaling initially. Mining transcriptomic profiling of ER+ primary tumors from TCGA and METABRIC cohorts, we found *TP53* mutant tumors exhibited significantly lower *ESR1* mRNA levels compared to *TP53* WT tumors (Fig. 3a). Decreased ER-α and phosphor-ER-α (pS118) protein levels were also discerned in *TP53* mutant primary tumors in the TCGA RPPA data set (Supplementary Fig. 3a). Notably, the inverse correlation between *TP53* mutations and ER-α expression was not restricted to a specific PAM50 tumor subtype, PR positivity, *TP53* mutation type, or the gain-of-function status^43^ of the mutant p53 protein (Supplementary Fig. 3b, c). In addition, *TP53* mutant tumors also showed dampened estrogen response signature (Hallmark Estrogen Response Early Signature) enrichment compared to *TP53* WT tumors in both cohorts (Fig. 3b), and again this was in general not associated with any particular contexts, except that the effect was more predominant in TCGA Luminal B tumors (Supplementary Fig. 3d, e). In summary, mutant p53 is associated with decreased ER-α expression and downstream ER activation in ER+ primary tumors, implicating that mutant p53 may block the ability of tumor cells to acquire *ESR1* mutation-induced hyperactivation or forces these cells to negate the necessity for acquisition of *ESR1* mutations and thus develop ER-independent survival machinery already in primary tumors, hence hinder subsequent double mutant co-occurrence.

To further elucidate genes driving this negative association between mutant p53 and ER signaling, we examined the expression difference of each individual estrogen response early signature gene (*n* = 200) between *TP53* mutant and WT tumors in TCGA and METABRIC. We identified a subset of 70 genes (p53-associated E2 response genes, *TP53*-ER Signature) that were consistently decreased in *TP53* mutant tumors in both cohorts, whereas another 60 genes constantly remained unchanged (non-p53-associated E2 response genes, Non-*TP53*-ER Signature) (Fig. 3c, d and Supplementary Table 2). As an independent validation, the *TP53*-ER Signature was markedly enriched in *TP53* WT breast cancer cell lines (*n* = 33) compared to *TP53* mutant lines (*n* = 9), whereas the Non-P53-ER Signature failed to differentiate the two subgroups, emphasizing the specificity of the defined gene sets (Supplementary Fig. 3f and Supplementary Table 3).

To link this finding to hyperactive *ESR1* mutations, we reproduced this analysis on metastatic tumors from MET500 and POG570 cohorts. Similar to the findings in primary tumors, *TP53* mutations exhibited an inverse correlation with *ESR1* mRNA expression in both cohorts in *ESR1* WT tumors (Fig. 3e). Applying the two p53-stratified E2 response signatures, we again observed that the *TP53*-ER Signature, but not the Non-*TP53*-ER Signature, differentiated *TP53* WT and mutant metastatic tumors (Fig. 3f). Importantly, enrichment levels of the *TP53*-ER Signature were higher than Non-*TP53*-ER Signature in *ESR1* mutant tumors but not *ESR1* WT counterpart (Fig. 3f), indicating that the p53-associated ER activation may be more important in the mutant ER hyperactivation state.

### Mutant p53 is linked to decreased ER expression and activation via loss of transactivation and a gain of miRNAs targeting ER

**Fig. 4:**
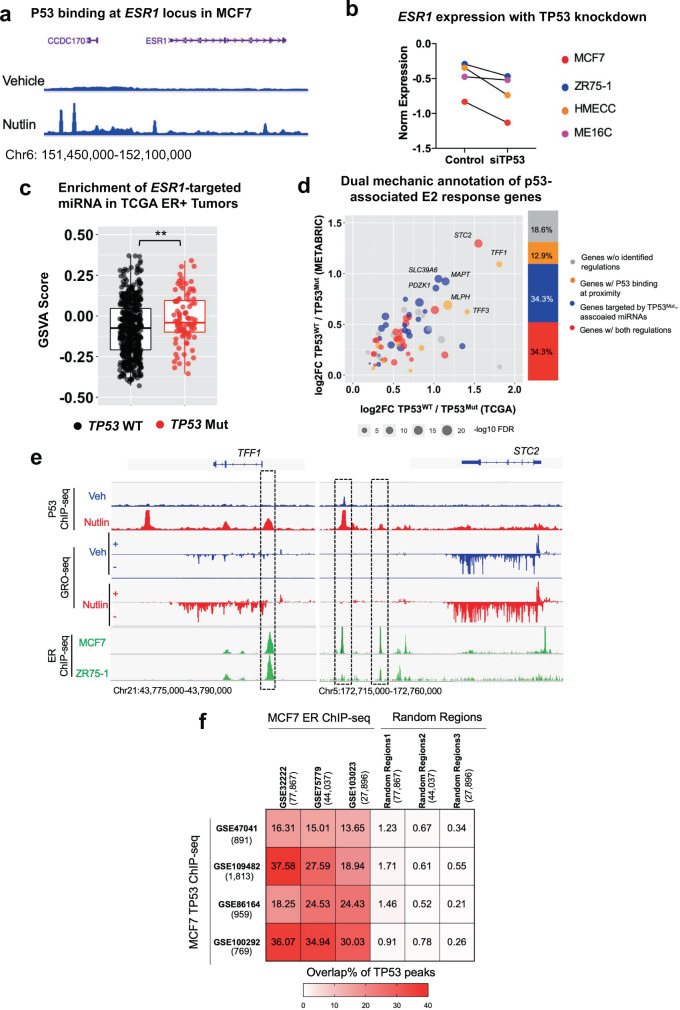
Mutant p53 links to ER repression via loss of transactivation and gain of ER-targeting miRNA. **a** Genomic track screen shot of WT p53 binding at *ESR1* locus before and after nutlin treatment in MCF7 cells. ChIP-seq data were obtained from GSE86164. **b** Line plot showing the expressional changes of *ESR1* before and after transient *TP53* knockdown for 36 h in four *TP53* WT ER+ cell lines. Data were downloaded from GSE3178. **c** Box plot representing the enrichment level of potential *ESR1*-targeting miRNA set in *TP53* WT (*n* = 457) versus *TP53* mutant (*n* = 87) ER+ primary tumors in TCGA cohort. Box plots span the upper quartile (upper limit), median (center) and lower quartile (lower limit). Whiskers extend a maximum of 1.5X IQR. Mann–Whitney U test (two-sided) was used. (**p < 0.01). **d** Scattered plot showing the correlation of the ratios between *TP53* WT/*TP53* Mut tumors of the 70 *TP53*-ER signature genes between TCGA and METABRIC ER+ tumors. Genes were classified into four groups indicating different association with WT p53 binding (WT p53 ChIP-seq annotated genes, *n* = 4356 in total) and/or mutant p53-regulated miRNA (Mutant p53 miRNA annotated genes, *n* = 10,316 in total). Top seven genes were specified with names. **e** Genomic track screen shot of WT p53 binding (MCF7), GRO-seq signal (MCF7) and ER binding (MCF7/ZR75-1) at *TFF1* (left panel) and *STC2* (right panel) gene locus. The former two data sets were indicated with or without nutlin treatment. Shared peaks between p53 and ER at proximity of these two genes were highlighted with frames. Data were downloaded from GSE86164, GSE53499, and GSE32222. **f** Heatmap depicting the overlap percentages of the four p53 ChIP-seq profiles with three independent ER ChIP-seq data sets from GSE32222, GSE75779, and GSE103023. Specific peak numbers of each profile were labeled with the GSE accession numbers. Fisher’s exact test (two-sided) was used to compare overlap ratio of each p53 binding profile with ER and the corresponding randomized regions of the same peak numbers.

To identify how mutant p53 compromises ER expression and its activity, we examined direct p53 binding by interrogating a p53 ChIP-seq data set in MCF7 cells which expresses WT p53^51^. P53 recruitment at four different genomic sites at the *ESR1* gene locus was detected after nutlin (a compound that blocks MDM2 to stabilize p53 protein) treatment (Fig. 4a). This is consistent with a previous study showing recruitment of p53 to the *ESR1* gene promoter using ChIP-qPCR in MCF7 cells^38^. Further, we identified decreased *ESR1* expression after p53 transient knockdown in two different p53 WT ER+ breast cancer and two other immortalized epithelial cell lines^52^ (Fig. 4b). Furthermore, leveraging two other public RNA-seq data sets^51,53^ showed increased expression of ER downstream target genes (e.g., *GREB1*, *IGFBP4*) upon nutlin treatment in MCF7 cells (Supplementary Fig. 4a). Overall, p53 serves as a direct transcriptional activator of the *ESR1* gene, which partially explains the decreased ER expression and downstream genes in p53 mutant tumors.

*ESR1* expression is known to be regulated by miRNAs^54–56^. To study this further, we identified 269 different miRNAs potentially targeting the *ESR1* transcript from miRbase^57^, and 89 of them showed a negative correlation with *ESR1* mRNA expression in TCGA ER+ tumors (Supplementary Fig. 4b, c and Supplementary Table 4). The overall abundance of those putative *ESR1*-targeting miRNA was higher in *TP53* mutant than WT tumors (Fig. 4c). This effect was more pronounced in tumors with missense and gain-of-function p53 mutations^43^ (Supplementary Fig. 4d). Of note, some of these mutant *TP53*-upregulated miRNAs have previously been shown to reduce ER expression in breast cancer models such as miR130b and miR301 (Supplementary Fig. 4e)^55,58,59^.

To further examine whether the putative dual-mechanism of *TP53* regulation of *ESR1* expression via both transcriptional regulation and via miRNA targeting is consistent with the previously identified 70 *TP53*-ER Signature genes, we annotated these genes as associated with “WT p53-binding sites” and/or “mutant p53-associated miRNAs”. To accomplish this, we 1) annotated genes associated with p53 ChIP peaks (−/+100 kb) from four independent MCF7 ChIP-seq data sets^51,53,60^ and 2) annotated the 89 *TP53* mutant tumor-associated upregulated miRNAs with their putative target genes. Among the 70 *TP53*-ER Signature genes, 33 (WT p53 transactivation) and 48 (mutant p53 miRNA-related) genes were identified. Overall, 57 (81.4%) of the *TP53*-ER Signature genes were annotated and 24 (34.3%) of them were linked to both mechanisms in the context of the MCF7 cell line (Fig. 4d). Proximal p53 binding sites were further visualized in MCF7 for two of the top consistently altered *TP53*-ER Signature genes-*TFF1* and *STC2*, showing notable transcriptional enhancement after nutlin treatment revealed by GRO-seq^61^ (Fig. 4e). Intriguingly, we found that some of the p53 binding sites overlapped with ER binding sites in two ER+ *TP53* WT cell lines (MCF7 and ZR75-1)^62^, suggesting p53 as a potential ER coregulator to facilitate ER downstream gene transactivation (Fig. 4e). Further intersection of the four p53 and three independent ER^62–64^ ChIP-seq profiles in MCF7 cell line confirmed that around 25% p53 binding sites co-localized with ER binding sites (Fig. 4f). In summary, the presence of mutant p53 may cause loss of genomic binding and enhance the expression of miRNAs to suppress ER expression and its downstream activity.

### Tumors with rare co-occurrence of *TP53* and *ESR1* mutations recapitulate the repression of ER activity by *TP53* mutation and exhibit unique immune features

**Fig. 5:**
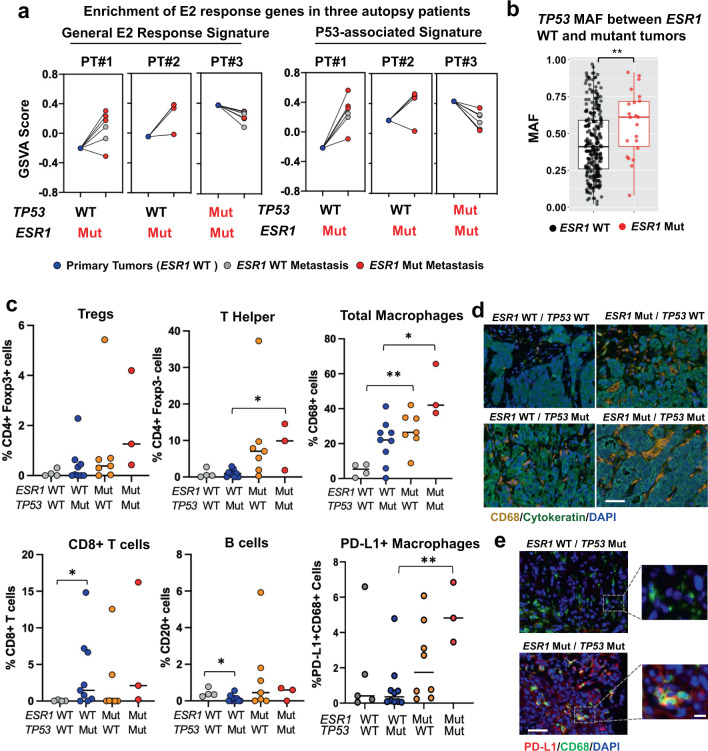
Tumors with rare co-occurrence of *TP53* and *ESR1* mutations recapitulate the repression of ER activity by *TP53* mutation and exhibit unique immune features. **a** Line plots showing the enrichment level alterations of general Estrogen Response Early signature (left panel) and *TP53*-ER Signature (right panel) from primary to each metastatic tumor of the three individual autopsy patients. Mutations status on specific specimens was indicated below. **b** Box plot showing the *TP53* mutation allele frequencies between *ESR1* WT and mutant tumors merged from MSKCC, POG570, MET500, INSERM, and METAMORPH cohorts (*ESR1* WT *n* = 266; *ESR1* Mut *n* = 22). Box plots span the upper quartile (upper limit), median (center), and lower quartile (lower limit). Whiskers extend a maximum of 1.5X IQR. Whitney U test (two-sided) was used. (**p < 0.01). **c** Dot plots representing the quantification of the abundance of five immune cell subtypes identified from multiplexed fluorescent staining from UC cohorts. Samples were separated based on *ESR1* and *TP53* genotypes (*ESR1* WT/*TP53* WT *n* = 5; *ESR1* WT/*TP53* Mut *n* = 10; *ESR1* Mut/*TP53* WT *n* = 8; *ESR1* Mut/*TP53* Mut *n* = 3). Numbers represent positive cells percentages of non-tumor cells (CK negative) from the field except PD-L1/CD68 dual staining, where number represents positive cells percentage of all cells in the corresponding filed. Median of each group was indicated. Whitney U test (two-sided) was applied for the comparisons between any of the two groups. (*p < 0.05; **p < 0.01). **d** Representative images showing total macrophages in tumors with different *ESR1* and *TP53* genotypes from UC cohort. CD68 (orange) is part of a multiplex IF containing panel including CD4 (yellow), Foxp3 (green), CD8 (magenta), CD20 (red), cytokeratin (teal), and DAPI (blue). Images were taken under 20× magnification. Scale bar = 50 μm. **e** Representative images showing PD-L1 + macrophages dual-IF staining on tumors with *TP53* mutation only and *ESR1*/*TP53* mutations. PD-L1 (red) and CD68 (green) were co-stained along with DAPI (blue). Images were taken under 20× magnification. Specific regions were further zoomed in to highlight target cells. Scale bar = 50 μm (left panel) and 5 μm (right panel).

Our analysis above suggests that acquisition of *ESR1* mutations may not be favorable or necessary in a tumor already harboring a *TP53* mutation. To test this hypothesis in the setting of co-occurrence, we interrogated a data set from a recent study by Siegel et al. where they conducted simultaneous DNA and RNA profiling on primary and multiple intra-patient paired metastatic tissues^65^. Among all 16 patients, we identified three ER+ cases with *ESR1* mutations including two *TP53* WT and one with co-occurrence of a *TP53* mutation. We then directly assessed the enrichment levels of a general E2 response signature and the *TP53*-ER Signature. As expected, both signatures were more enriched in the 7/8 metastatic tumors with *ESR1* mutation with WT *TP53* (PT#1 and PT#2), recapitulating the ER signaling enhancement conferred by *ESR1* mutations during metastatic development (Fig. 5a). Notably, the *TP53*-ER Signature was also increased in two of the *ESR1* WT *TP53* WT metastatic lesions in patient #1 likely due to gain of ER expression as an alternative mechanism to enhance ER activity (Supplementary Fig. 5). In contrast, the enrichment of both signatures was reduced in all five metastatic tissues from PT#3, which all harbored *TP53* mutations including two *ESR1* mutant and three *ESR1* WT tumors in PT#3. In line with this, we found that *TP53* mutation allele frequencies were significantly higher in tumors with both mutations than *TP53* mutant *ESR1* WT tumors in merged metastatic tumor cohorts (Fig. 5b), suggesting a higher degree of p53 functional impairment is required to sufficiently block ER signaling in the presence of *ESR1* mutations. Together, these results indicate that *TP53* mutations may abrogate the advantages of ER constitutive activation instigated by hotspot *ESR1* mutations.

Lastly, since both mutations are reported to influence tumor immune landscapes, we examined the immune features of dual-mutant tumors making use of our recent study with multiplexed immune cell subtype staining of 26 metastatic tumors in the UC cohort^23^. We observed differential immune-modulatory effects attributed by *TP53* or *ESR1* mutations (Fig. 5c). CD8+ T cells were more abundant in *TP53* mutant tumors, whereas macrophages were enriched in tumors with either mutant (Fig. 5c). Of note, dual-mutant tumors showed a more pronounced increase in total and PD-L1 positive macrophage population (Fig. 5c–e).

## Discussion

**Fig. 6:**
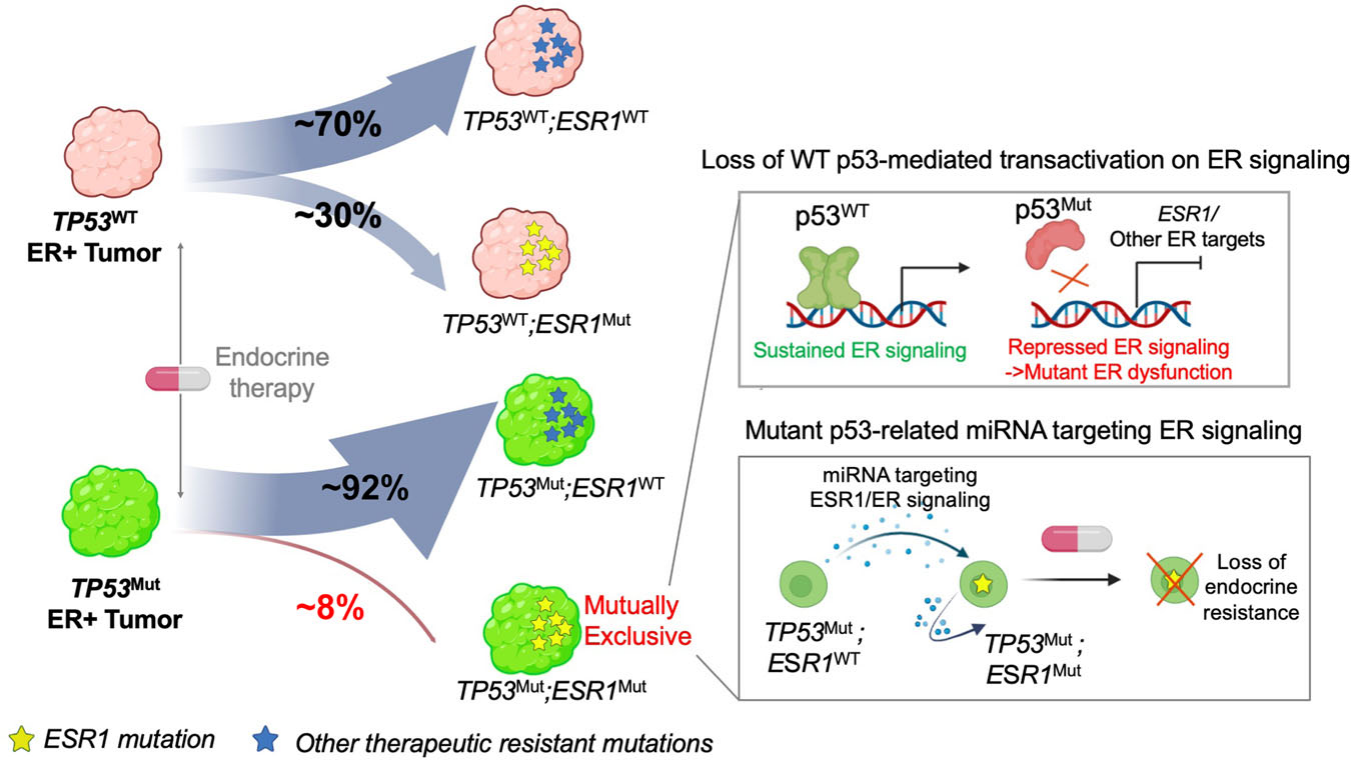
Schema of proposed mechanism of *TP53*-*ESR1* mutation mutual exclusivity in ER+ metastatic breast cancer. In the scenario of *TP53* mutations as the primary driver, ER signaling is disrupted by (1) loss of WT p53 transactivation and (2) mutant p53-regulated miRNA. Thus *ESR1* mutations are less frequently gained in *TP53* mutant tumors. In the case of a non-*TP53* mutation serving as the founder, clones acquiring *ESR1* mutations could efficiently outgrow under endocrine therapy and result in *ESR1* mutant-dominated progression.

Epistatic relationships between two mutations may yield sophisticated insight into biological cooperation or antagonism. In the present study, we explored the potential mechanisms underpinning the mutual exclusivity between *ESR1* and *TP53* mutations in ER+ breast cancer. The results of our hypothesis-generated study suggest a unidirectional inhibitory effect from mutant p53 upon ER signaling, which may preclude a selective advantage acquired *ESR1* mutations in a tumor with mutant p53 as the founder mutation. In contrast, in the absence of *TP53* mutation, acquired *ESR1* mutations may play a predominant role under the selective pressure of endocrine therapy, particularly aromatase inhibitors that block production of estrogen, giving rise to *ESR1* mutation-enriched metastatic lesions in approximately 30% of ER + metastatic breast cancers (Fig. 6).

Our bioinformatic analysis based upon data from metastatic tumors did not suggest any obvious impact of *ESR1* mutations on p53 signaling activity. However, several pre-clinical studies have shown that ER can either activate or suppress p53 activity^34,35,66^. A possible explanation is that these earlier studies largely relied on a limited number of ER+ breast cancer cell models (e.g., MCF7 and ZR75-1), which do not fully represent the extent of inter-patient heterogeneity. Further investigation using other *TP53* WT ER + in vivo and in vitro models are required. Furthermore, it is also likely that mutant ER has gained activities that go beyond that of ligand-independent activities of WT ER. Recent omic studies by us and others^23–25,67,68^ showed a large number of *de novo* gained or lost genomic binding events and transcriptomic/epigenetic regulation of *ESR1* mutations compared to WT-ER activated by E2, potentially losing the regulatory potential towards key p53 binding sites.

Furthermore, our finding of the positive regulation between WT p53 and ER signaling pointed out a potential interplay between p53 activation and endocrine treatment response. It is possible that a subpopulation of cells may have potentiated ER signaling following the p53 activation by first-line chemotherapy, which makes them more sensitive towards a subsequent endocrine therapy. This is in line with the recent clinical observation that *TP53* mutations are largely associated with endocrine resistance in ER+ breast cancer^69,70^, suggesting that a functional p53 is a favorable predictive factor for endocrine therapy. In addition, previous clinical trials showed that chemo-endocrine combination as first-line therapy improved outcomes, as exemplified by aromatase inhibitors plus capecitabine for postmenopausal women^71^ and tamoxifen plus CAF (cyclophosphamide, doxorubicin, and fluorouracil) for premenopausal women^72^. Lastly, a recent pre-clinical study showed improved fulvestrant response when combined with MDM2 inhibitor to activate p53 in ER+ breast cancer models in vitro and in vivo^73^. In summary, our findings support a possible rationale that p53 activation might be synergistic with endocrine therapy.

Our mechanistic exploration highlighted mutant p53-associated miRNA regulation as an indirect way to block ER activity. Previous global miRNA profiling suggested that mutant p53 has a suppressive role on miRNA production. It has been reported that WT p53 regulates the processing of precursor miRNAs via either directly binding to DROSHA or maintaining DICER1 expression, whereas mutant p53 might disrupt these positive regulations^74–76^, and DICER1 protein is low in triple-negative breast cancer^77^. However, recent studies showed induction of specific miRNAs such as miR-128-2 and miR-155, associated with GoF mutant p53^78–80^. Consistent with this, our data also suggested a more pronounced *ESR1*-targeting miRNA enrichment in tumors with GoF mutant p53. It is possible that mutant p53 tumors selectively elevate specific miRNAs targeting *ESR1* transcript regardless of the repressive regulation on other miRNA networks. Furthermore, these miRNAs might not be directly regulated by mutant p53 but other genetic events prevalently co-occurring with *TP53* mutations. For instance, the frequent co-occurrence of MYC amplifications with *TP53* mutations in breast cancer might also lead to aberrant miRNA network activation^81,82^ and could partially explain the discrepancy between our findings from clinical specimens and previous results from cell models discussed above. Importantly, while the loss of WT p53 transactivation suggests a potential intra-clonal suppression mechanism, the miRNA-based modulation might represent an inter-clonal inhibitory machinery from *TP53* mutant clones towards different clones harboring *ESR1* mutations. In addition, it is plausible that *ESR1* and *TP53* mutations are functionally redundant, hence the suppressive effect on ER signaling drives *TP53* mutant cells to grow in an ER-independent manner. Further molecular and cellular experimental investigations are warranted to test these hypotheses further.

Previous studies have shown immune modulation in both *TP53* and *ESR1* mutant tumors^23,83–85^. First, it has been reported that specific p53 mutant variants such as R175H are associated with elevated macrophage recruitment^86,87^. In addition, Minin et al. recently reported that mutant p53 fuels NF-kB activation by inhibition of DAB2IP and thus triggers inflammatory stimulation^88^. Research linking *ESR1* mutations and immune activation remains scarce. We reported previously that *ESR1* mutant metastatic samples have higher levels of macrophages, and also proposed possible mechanisms including activation of innate immune response via STING pathways and production of CHI3L1 enhanced recruitment of macrophages via elevated S100A8/A9-TLR4 signaling^23,83^. Here we identified a further significantly increased level of total and PD-L1 positive macrophages in the rare tumors with co-occurring *TP53*-*ESR1* mutations. Although this finding suggests that co-occurring *ESR1* and *TP53* mutations might alter immune infiltration in a synergistic manner, providing an alternative explanation for the mutual exclusivity, our findings are limited by small sample numbers, and thus need to be interpreted with caution. Limited number of samples used in the transcriptomic data analysis clearly warrants additional bioinformatic investigation using larger cohorts of metastatic disease once those become available. Furthermore, our mechanistic exploration regarding p53 binding is restricted to the MCF7 cell line model due to the limited available data sets. This single model is a limited representation of a highly heterogenous patient population and thus further evaluations in additional *TP53* WT ER+ cell models are required. Another limitation of our study is the lack of validation of proposed mechanisms in pre-clinical in vitro and in vivo models, but we strongly believe that our work generated an in-depth and testable hypothesis regarding mutational exclusivity of *ESR1* and *TP53* in metastatic ER+ breast cancer, ultimately paving a path for future therapeutic design based on these insights.

## Methods

### Mutation analysis from publicly available data sets

Sources of *ESR1* and *TP53* mutation annotation results from different cohorts are specified in “Data Availability” section. For the mutual exclusive analysis, only ER+ metastatic samples are selected from each cohort.

Odds ratios were calculated by the equation of *Odds ratio* = *[n(TP53 WT ESR1 WT)/ n(TP53 Mut ESR1 WT)]/[n(TP53 Mut ESR1 WT)/n (TP53 Mut ESR1 Mut)]*. Upper and lower 95% confidence interval were further calculated to represent the expected range of odds ratio. Fisher’s exact test was used to compute the *p* value. A odds ratio below 1 typically represents a trend of mutual exclusivity and vice versa for odds ratio above 1.

Mutant p53 loss-of-function scores (LOFS) were calculated based on MU*TP53*LOAD (Mutant *TP53* Loss Of Activity Database) from “The *TP53* Website”^89^. Average transactivation percentage (normalized to WT p53) from eight canonical p53 target gene promoters (WAF1, MDM2, BAX, 14-3-3-s, AIP, NOXA, p53R2) was calculated for each missense mutant variant based on the experiments documented in the data base. *TP53* missense variants were then categorized into three subsets based on Loss-of-function scores: weak (LOFS > 10, p53 variant transcriptional activity is above 10% of WT p53, *n* = 50); medium (1 < LOFS < 10, p53 variant transcriptional activity is between 1 and 10% of WT p53, *n* = 42) and strong (LOFS < 1, p53 variant transcriptional activity is below 1% of WT p53, *n* = 21). Of note, mutations other than missense variants (e.g., nonsense mutations, INDELs) were not included in this classification as no data were recorded in the database. The full list of mutations of these three categories is provided in Supplementary Table 5.

*TP53* gain-of-function missense and other missense variants were divided based on a previous publication^43^. Specifically, GoF *TP53* missense mutations were defined as previously validated and reported *TP53* missense mutations with gain-of-function. Other *TP53* missense mutations were defined as non-GoF mutations or uncharacterized *TP53* missense mutations. Full list of mutations of these two categories is provided in Supplementary Table 6.

### Metastatic breast cancer patient samples from UC cohort

Mutation analysis was done as previously described^90^. Core needle biopsies were acquired from patients, who gave their informed written consent, with ER+/Her2- measurable or evaluable metastatic breast cancer (MBC) without CNS disease enrolled in clinical trial NCT02953860. Research on tissues from the trial is covered under IRB protocol COMIRB 16-1001. Median age of patients was 61 years (46-87); PS 1 (0-1); a median of 2 prior chemotherapy and 2 prior hormonal therapies for metastatic disease (including 7 with prior Fulvestrant), and 90% had visceral disease. Formalin fixed paraffin-embedded sections were analyzed for mutations in *ESR1* exon 8 as well as 67 other gene hotspots frequently altered in cancer using a modified Archer VariantPlex Solid Tumor Assay through the CMOCO Laboratory (University of Colorado Department of Pathology).

### P53 immunohistology and scoring

Five micron thick paraffin sections were prepared for immunodetection of p53 (Cell Marque, Rocklin, CA; #453M-94; 1:500). Antigens were revealed in pH 9.5 BORG solution (Biocare Medical, Concord, CA) for 10 min at 110 °C (NxGen Decloaker, Biocare) with a 10 min ambient cool down. Immunodetection of p53 was performed on the Benchmark XT autostainer (Ventana Medical Systems, Roche, Indianapolis, IN) with primary incubation for 32 min using UltraView DAB polymer detection (Ventana) at 37 °C. All sections were counterstained in Harris hematoxylin for 2 min, blued in 1% ammonium hydroxide, dehydrated in graded alcohols, cleared in xylene and coverglass mounted using synthetic resin. Negative controls to confirm the specificity of the immunostaining included omission of the primary antibody incubation step in the IHC protocol and substitution with the primary antibody diluent.

P53 by immunohistochemical analysis was evaluated by identifying the percentage of tumor with positive nuclear expression for p53 and results were interpreted as follows: Tumor with staining between 0 and 15% was considered null aberrant (positive result) and tumor with staining between 80 and 100% was considered positive aberrant (positive result). Tumor with staining between 16 and 79% was considered to be wildtype (negative result).

### Multiplexed fluorescence staining

FFPE sections were stained for immune markers using multiplex Opal™ TSA technology (Akoya Biosciences) along with the Vectra 3 Automated Quantitative Pathology Imaging System. TIL antibodies used were: CD4 (Agilent Cat# M7310, RRID:AB_2728838, 1.4 μg/ml), Foxp3 (Abcam Cat# ab20034, RRID:AB_445284, 1:200), CD8 (Agilent Cat# M7103, RRID:AB_2075537, 0.4 μg/ml), CD20 (Abcam Cat# ab9475, RRID:AB_307267, 1:300), and CD68 (Agilent Cat# GA60961-2, RRID:AB_2661840, 0.12 μg/ml), and pan cytokeratin (Agilent Cat# M3515, RRID:AB_2132885, 0.18 μg/ml) was used to identify tumor epithelium. PD-L1/CD68 Co-IF antibodies used were: PD-L1 (Abcam Cat# ab228462, RRID:AB_2827816, 1:400) and CD68 (Agilent Cat# GA60961-2, RRID:AB_2661840, 0.12 μg/ml). Dapi (Akoya Cat# FP1490) was used as a counterstain for each core needle biopsy, and positive cells in three to five 669 μm × 500 μm fields were scored using InForm software (Perkin Elmer) using either a pixel or cell-based algorithm including both tissue and cell segmentation.

### Transcriptomic and miRNA-sequencing analysis

For the MET500 cohort^40^, transcript counts from all samples were quantified with Salmon v.0.8.2 and converted to gene-level counts with tximport. The gene-level counts from all studies were then normalized together using TMM with edgeR. Log2 transformed TMM-normalized counts per million [log_2_(TMM-CPM + 1)] were used for analysis. To predict ER positivity based on *ESR1* expression, the TCGA cohort was used as a reference. Briefly, putative “ER+” (higher than a pre-defined cutoff) and “ER-” (lower than a pre-defined cutoff) statuses were predicted based on *ESR1* log_2_(CPM+1) values of 1045 primary tumors using each consecutive interval of 0.1 between 3 (first quartile of *ESR1* expression levels in MET500) and 8.8 (third quartile of *ESR1* expression levels in MET500). The predicted results were then compared to pathological ER status identification for each cutoff selection. Log_2_(CPM+1) values of 5.6 were determined as the final cutoff for ER status due to a highest concordance ratio towards pathological records (95.5%). 46 putative ER-positive samples were then filtered in the MET500 cohort.

For the POG570 cohort^41^, ER status of each patient was additionally requested from the cited original resources. Log_2_(CPM+1) values were used for downstream analysis.

TCGA RNA-seq reads were reprocessed using Salmon v0.14.1^91^ and Log2 (TPM+1) values were used. TCGA RPPA and miRNA-seq data were directly downloaded from FireBrowse.

For the METABRIC data set, normalized probe intensity values were obtained from Synapse. For genes with multiple probes, probes with the highest inter-quartile range (IQR) were selected to represent the gene.

For pan-breast cancer cell line transcriptomic analysis, 97 breast cancer cell line RNA-seq data were reprocessed using Salmon and merged from three studies^92–94^. Cell lines with *ESR1* Log2 (TPM+1) above 3 were selected for further signature analysis. *TP53* mutation data were obtained from Expasy data base^95^.

For in vitro ER+ cell line *TP53* knockdown microarray data^52^ and nutlin-treated MCF7 RNA-seq data sets^51,53^, raw counts were normalized using TMM with edgeR. Log2 transformed TMM-normalized counts per million [log_2_(TMM-CPM+1)] were used for *GREB1* and *IGFBP4* expression comparison. Gene set variation analyses were performed using the GSVA package^96^ with selected gene sets. To select potential *ESR1* transcript targeting miRNA in clinical samples, miRNAs with matched anti-*ESR1* sequence were first obtained from miRbase and then matched to the processed TCGA miRNA-seq data sets. Specific miRNAs showing negative trend of correlation (*R* < 0) with *ESR1* expression were further selected for enrichment analysis. Full list of 89 selected *ESR1*-targeting miRNAs can be found in Supplementary Table 4.

### ChIP-sequencing analysis

Processed p53 ChIP-seq data were directly downloaded in BED format from Cistrome DB which uniformly aligns all the raw reads to hg38 references genome and calls binding peaks^97^. Original *TP53* ChIP-seq sources are indicated in the Data Availability section. Genes locate at −/+100 kb of p53 bindings sites were annotated using CistromeDB Toolkit. The four data sets were selected based on the criteria of predicted target genes above *N* = 1000 and the union of these identified targeted genes were used for subsequent integrative analysis. P53 ChIP-seq and GRO-seq with nutlin treatment were visualized on WashU Epigenome Browser^98^. For ER and p53 ChIP-seq intersection analysis, random peak sets generation was conducted using regioneR package^99^. Peak overlap was performed using DiffBind^100^. Original sources are indicated in the “Data Availability” section.

### Statistical analysis

GraphPad Prism software version 7 and R version 3.6.1 were used for statistical analysis.

### Reporting summary

Further information on experimental design is available in the Nature Research Reporting Summary linked to this paper.

## Supplementary information

Supplementary Information

Reporting Summary Checklist

## Data Availability

All data analyzed in this study have previously been reported and are publicly available: *ESR1* and *TP53* mutation annotation results of MSKCC, INSERM, TCGA, and METABRIC cohorts were directly downloaded from cBioPortal (https://www.cbioportal.org/)^101^. Mutation data from POG570^41^ and MET500^40^ cohorts were obtained from the specific web-portals (https://met500.path.med.umich.edu) and (https://www.bcgsc.ca/downloads/POG570/), and mutation matrix from METAMORPH and samples from the UNC Rapid Autopsy program were obtained from the original publications^42,65^. “UC” is our in-house cohort and mutations were called as previously described^23^. For the MET500 cohort^40^, RNA-seq fastq files from 91 metastatic breast cancer samples were downloaded from the Database of Genotypes and Phenotypes (dbGaP) with accession number phs000673.v2.p1. For the POG570 cohort^41^, raw count matrixes and mutation statuses were downloaded from the BCGSC portal. RNA-seq data and clinical information from TCGA and METABRIC were obtained from the GSE62944 and Synapse software platform under accession number syn1688369 respectively. TCGA RPPA and miRNA-seq data were directly downloaded from FireBrowse (http://firebrowse.org/). For pan-breast cancer cell line transcriptomic clustering, 97 breast cancer cell line RNA-seq data were merged from three studies^92–94^. In vitro ER+ cell line *TP53* knockdown microarray data were obtained from GSE3178^52^. Nutlin-treated MCF7 RNA-seq data sets were downloaded from GSE47042^53^ and GSE86221^51^. Original *TP53* ChIP-seq data were released from GSE109482 (deposited but not published), GSE86164^51^, GSE100292^60^ and GSE47041^53^. P53 ChIP-seq and GRO-seq with nutlin treatment were obtained from GSE86164^51^ and GSE53499^61^. ER ChIP-seq used for track visualization from MCF7 and ZR75-1 were obtained from GSE32222^62^. Other ER ChIP-seq data for intersection analysis were downloaded from GSE75779^64^ and GSE103023^63^.
