# Supplementary material for: Mutual exclusivity of ESR1 and TP53 mutations in endocrine resistant metastatic breast cancer
Source: NPJ Breast Cancer. 2022 May 10;8:62. doi: 10.1038/s41523-022-00426-w (PMC9090919; doi:10.1038/s41523-022-00426-w)
Supplement: Supplementary file 1 — Supplementary Information [file 41523_2022_426_MOESM1_ESM.pdf]

## Supplementary Figure 1

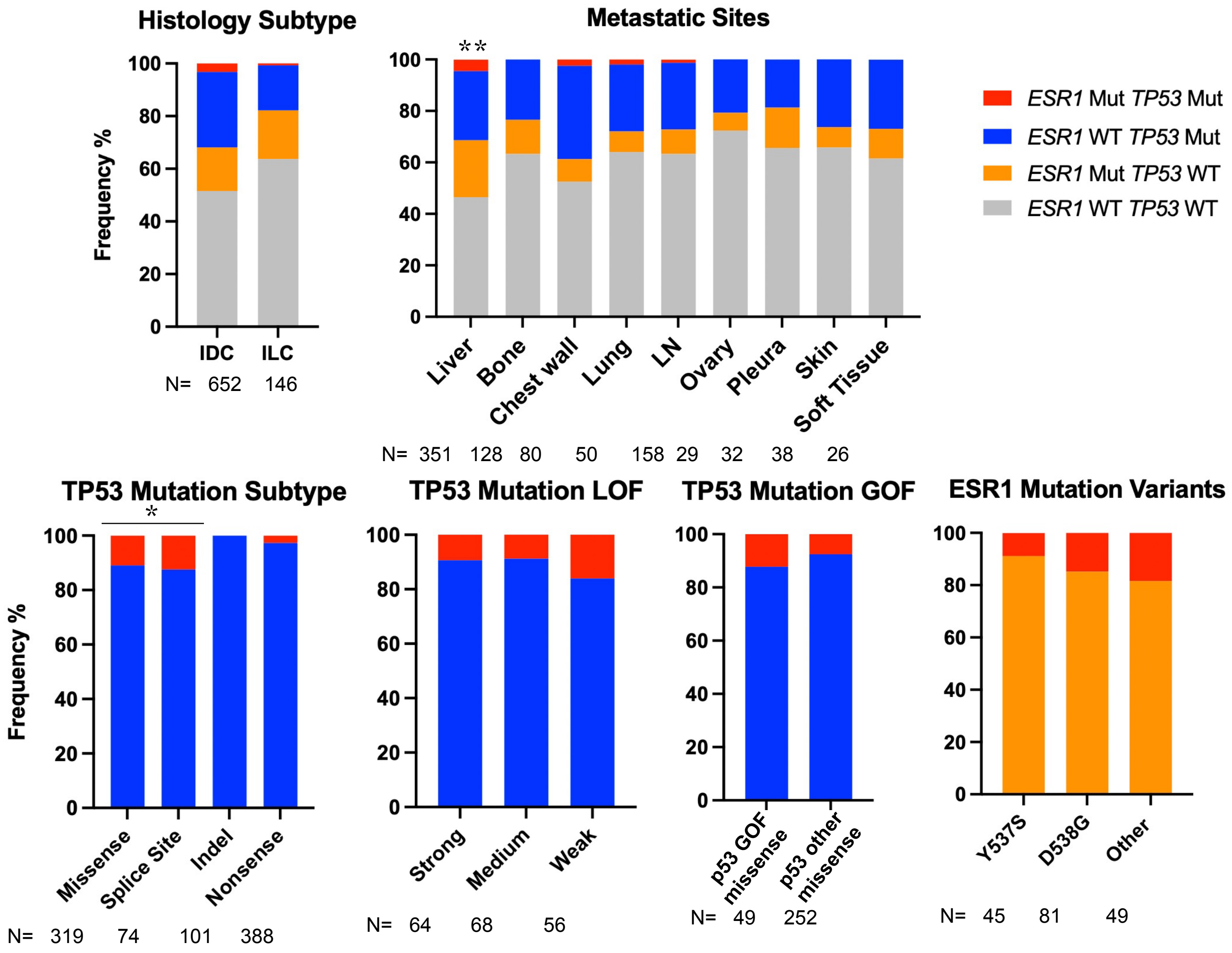


**Supplementary Figure 1. Distribution of *ESR1* and *TP53* mono- or dual-mutant tumors within different settings.**

Stacked bar plots depicting the frequencies of metastatic tumors with different genotypes: *ESR1* WT/ *TP53* WT (grey) *; ESR1* WT/ *TP53* Mut (blue) *; ESR1* Mut/ *TP53* WT (orange) *; ESR1* Mut/ *TP53* Mut (red) combined from six cohorts. Total numbers of each category are labelled below. Fisher's exact test (two-sided) was used to calculate enrichment of dual-mutant vs non-dual-mutant tumors in the corresponding categories. (* $p<0.05$; ** $p<0.01$)

# Supplementary Figure 2

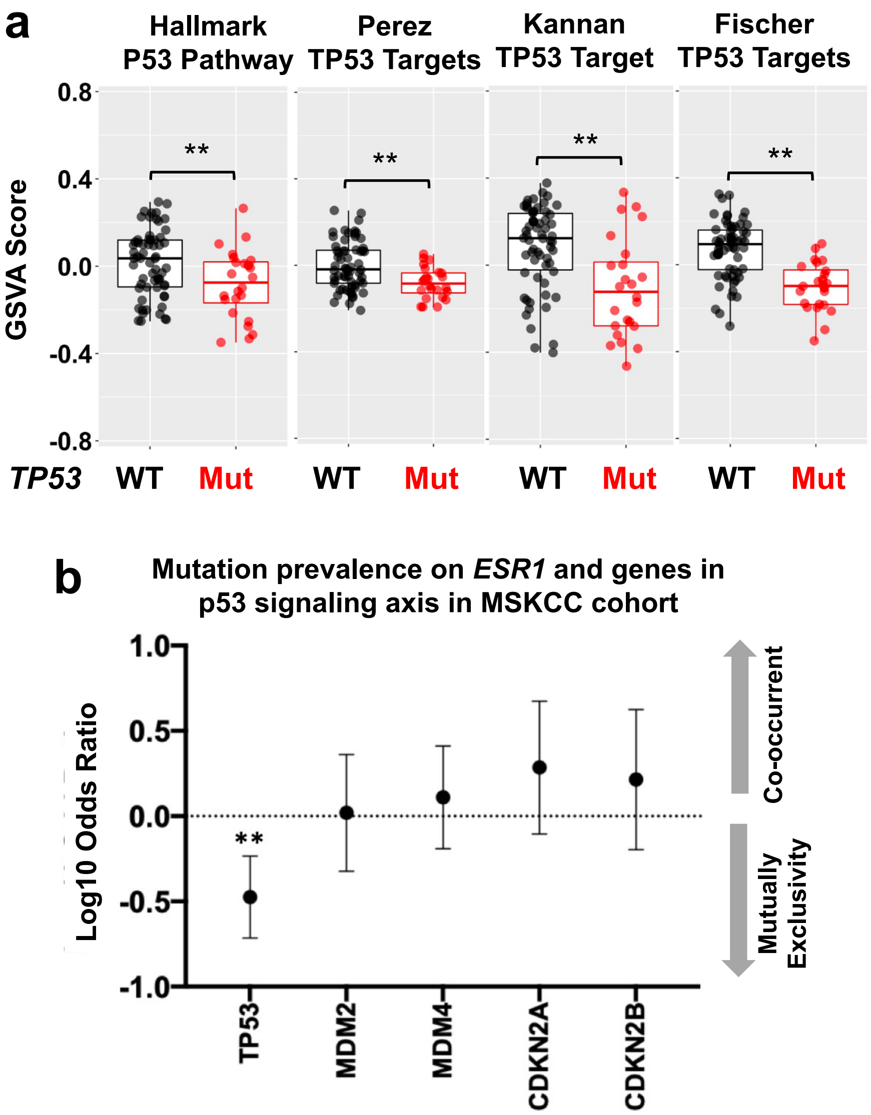


**Supplementary Figure 2. Validation of *TP53* signatures and *ESR1* mutation is solely exclusive with mutations on *TP53* but not other genes in *TP53* pathway.**

a. Box plots representing the enrichment levels of four different p53-associated gene signatures in TCGA cohort between *TP53* WT and mutant tumors. Box plots span the upper quartile (upper limit), median (centre) and lower quartile (lower limit). Whiskers extend a maximum of 1.5X IQR. Samples were randomized for 62 *TP53* WT and 24 *TP53* mutant tumors to keep the same statistic power as Fig. 2d. Mann Whitney U test (two-sided) was used. (** p<0.01).

b. Forest plot representing the odds ratio of *ESR1* mutation and genomic alterations on *TP53*, *MDM2, MDM4, CDKN2A* and *CDKN2B* in the MSKCC cohort. Fisher's exact test (two-sided) was used to test each combination. Log10 Odds ratio with 95% confidence interval is shown. (*p<0.05; ** p<0.01)

# Supplementary Figure 3

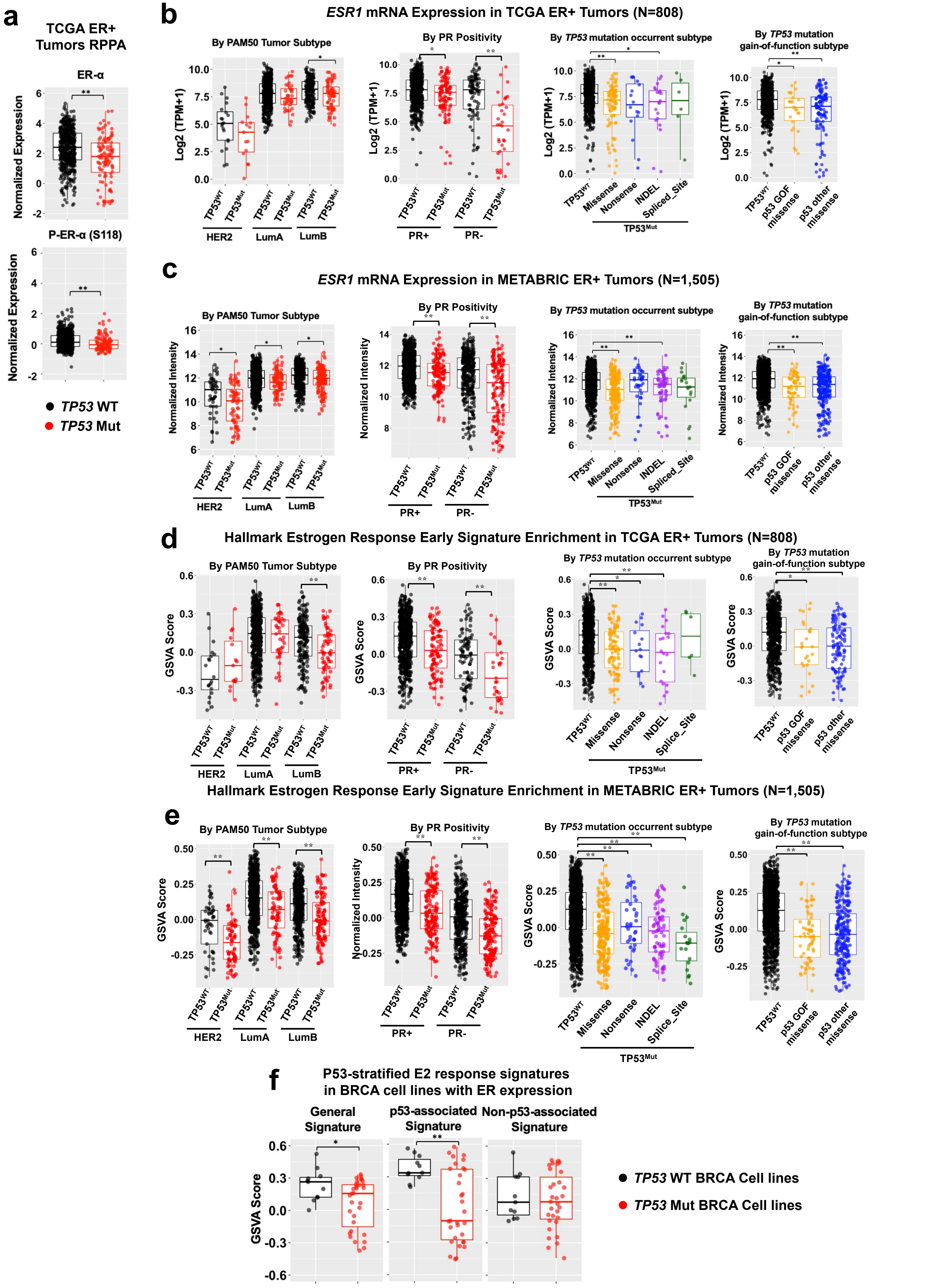

# Supplementary Figure 3-Continued

**Supplementary Figure 3. *TP53* mutation is inversely correlated to expression of ER-α and a subset of downstream genes.**
a. Box plots representing the protein level of total estrogen receptor-α (left panel) or phosphorylated estrogen receptor-α (Ser118) in *TP53* WT (n=516) versus *TP53* mutant (n=111) ER+ primary tumors from TCGA cohorts. Box plots span the upper quartile (upper limit), median (centre) and lower quartile (lower limit). Whiskers extend a maximum of 1.5X IQR. Mann-Whitney U test (two-sided) was used (** $p<0.01$).
b to e. Box plots showing *ESR1* mRNA expression (b&c) or Estrogen Response Early Signature Enrichment (d&e) in *TP53* WT vs *TP53* mutant ER+ tumors in different PAM50 subtypes; PR positive or negative contexts, different *TP53* mutation occurrent subtypes or *TP53* gain-of-function mutation subtypes. Analysis was performed separately in TCGA (b&d) and METABRIC cohorts (c&e). Box plots span the upper quartile (upper limit), median (centre) and lower quartile (lower limit). Whiskers extend a maximum of 1.5X IQR. Mann-Whitney U test (two-sided) was for each comparison (*$p<0.05$; ** $p<0.01$).
f. Box plots representing the enrichment levels of general “Estrogen Response Early” signatures, P53-ER Signature and Non-P53-ER Signature between *TP53* WT (n=11) and mutant (n=31) ER+ breast cancer cell lines. Box plots span the upper quartile (upper limit), median (centre) and lower quartile (lower limit). Whiskers extend a maximum of 1.5X IQR. Mann-Whitney U test (two-sided) was for each comparison (*$p<0.05$; ** $p<0.01$).

# Supplementary Figure 4

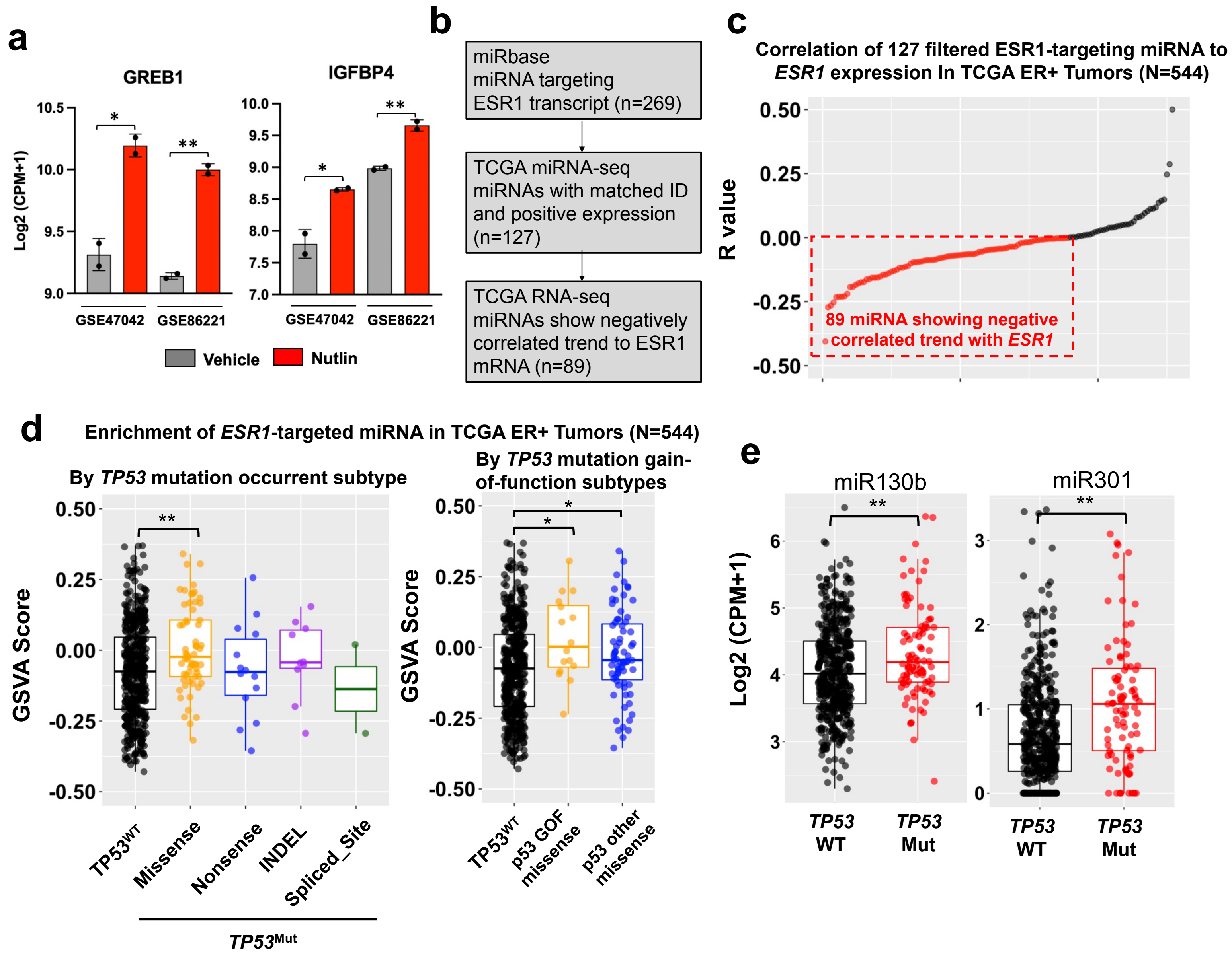


**Supplementary Figure 4. Mutant p53 links to ER repression via loss of transactivation and gain of ER-targeting miRNA**

a. Bar plots showing two ER downstream target gene expression in MCF7 cells after nutlin treatment from two publicly available RNA-seq data sets (GSE47042 and GSE86221). Mean ± SD were shown for two replicates of each data set. Student's t test (two-sided) was used for each comparison.

b. Flow chart showing the procedure to select potential *ESR1*-targeting microRNA in TCGA ER+ tumors.

c. Dot plot highlighting the selected miRNA set with a negative trend of correlation with *ESR1* mRNA.

d. Box plots showing the enrichment levels of the potential *ESR1*-targeting microRNA set in *TP53* WT vs *TP53* mutations with different mutation occurrent subtypes (left panel) or gain-of-function subtypes (right panel). Box plots span the upper quartile (upper limit), median (centre) and lower quartile (lower limit). Whiskers extend a maximum of 1.5X IQR. Mann-Whitney U test (two-sided) was for each comparison (*p<0.05; ** p<0.01).

e. Box plots showing the abundance of two previously validated *ESR1*-targeting miRNA (miR130b/miR301) between TCGA *TP53* WT (n=457) and mutant (n=87) ER+ tumors. Box plots span the upper quartile (upper limit), median (centre) and lower quartile (lower limit). Whiskers extend a maximum of 1.5X IQR. Mann-Whitney U test (two-sided) was for each comparison (** p<0.01).

# Supplementary Figure 5

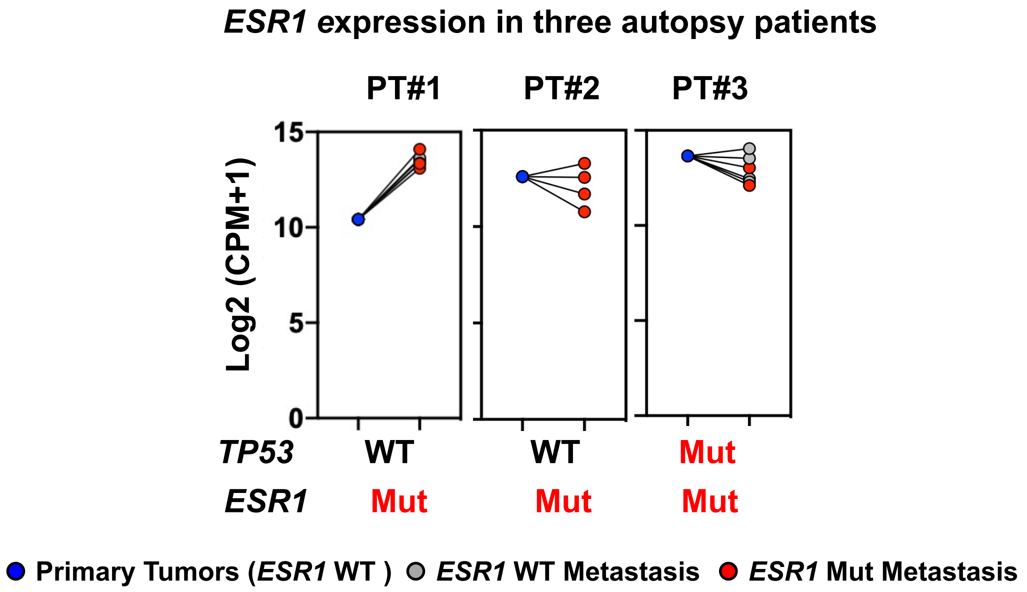


**Supplementary Figure 5. Rare cases of *TP53-ESR1* mutations co-occurrence recapitulate the repression on ER activity by *TP53* mutations.**
Line plots showing the *ESR1* mRNA expressional alterations from primary to each metastatic tumor of the three individual autopsy patients. Mutations status on specific specimens is indicated below.

**Supplementary Table 1**

| All Samples (n=1,056) | | | | *TP53* Missense Only (n=946) | | |
|---|---|---|---|---|---|---|
| | *TP53* WT | *TP53* Mut | | | *TP53* WT | *TP53* Mut |
| *ESR1* WT | 594 | 276 | | *ESR1* WT | 594 | 170 |
| *ESR1* Mut | 161 | 25 | | *ESR1* Mut | 161 | 21 |
| IDC Only (n=652) | | | | *TP53* INDEL Only (n=806) | | |
| | *TP53* WT | *TP53* Mut | | | *TP53* WT | *TP53* Mut |
| *ESR1* WT | 336 | 187 | | *ESR1* WT | 594 | 51 |
| *ESR1* Mut | 108 | 21 | | *ESR1* Mut | 161 | 0 |
| ILC Only (n=146) | | | | *TP53* Nonsense Only (n=792) | | |
| | *TP53* WT | *TP53* Mut | | | *TP53* WT | *TP53* Mut |
| *ESR1* WT | 93 | 25 | | *ESR1* WT | 594 | 36 |
| *ESR1* Mut | 27 | 1 | | *ESR1* Mut | 161 | 1 |
| Bone Metastasis Only (n=132) | | | | *TP53* Splice Site Only (n=779) | | |
| | *TP53* WT | *TP53* Mut | | | *TP53* WT | *TP53* Mut |
| *ESR1* WT | 81 | 31 | | *ESR1* WT | 594 | 21 |
| *ESR1* Mut | 20 | 0 | | *ESR1* Mut | 161 | 3 |
| Chest Wall Metastasis Only (n=80) | | | | *TP53* Strong LOS Only (n=808) | | |
| | *TP53* WT | *TP53* Mut | | | *TP53* WT | *TP53* Mut |
| *ESR1* WT | 42 | 29 | | *ESR1* WT | 594 | 47 |
| *ESR1* Mut | 7 | 2 | | *ESR1* Mut | 161 | 6 |
| Liver Metastasis Only (n=351) | | | | *TP53* Medium LOS Only (n=823) | | |
| | *TP53* WT | *TP53* Mut | | | *TP53* WT | *TP53* Mut |
| *ESR1* WT | 163 | 94 | | *ESR1* WT | 594 | 62 |
| *ESR1* Mut | 78 | 16 | | *ESR1* Mut | 161 | 6 |
| Lung Metastasis Only (n=50) | | | | *TP53* Weak LOS Only (n=822) | | |
| | *TP53* WT | *TP53* Mut | | | *TP53* WT | *TP53* Mut |
| *ESR1* WT | 30 | 15 | | *ESR1* WT | 594 | 58 |
| *ESR1* Mut | 4 | 1 | | *ESR1* Mut | 161 | 9 |
| Lymph Node Metastasis Only (n=159) | | | | *ESR1* D538G Mut Only (n=948) | | |
| | *TP53* WT | *TP53* Mut | | | *TP53* WT | *TP53* Mut |
| *ESR1* WT | 100 | 42 | | *ESR1* WT | 594 | 276 |
| *ESR1* Mut | 15 | 2 | | *ESR1* Mut | 66 | 12 |
| Ovary Metastasis Only (n=29) | | | | *ESR1* Y537S Mut Only (n=915) | | |
| | *TP53* WT | *TP53* Mut | | | *TP53* WT | *TP53* Mut |
| *ESR1* WT | 20 | 7 | | *ESR1* WT | 594 | 276 |
| *ESR1* Mut | 2 | 0 | | *ESR1* Mut | 41 | 4 |
| Pleura Metastasis Only (n=32) | | | | *ESR1* E380Q Mut Only (n=889) | | |
| | *TP53* WT | *TP53* Mut | | | *TP53* WT | *TP53* Mut |
| *ESR1* WT | 21 | 6 | | *ESR1* WT | 594 | 276 |
| *ESR1* Mut | 5 | 0 | | *ESR1* Mut | 17 | 2 |
| Skin Metastasis Only (n=38) | | | | *TP53* GOF Missense (n=804) | | |
| | *TP53* WT | *TP53* Mut | | | *TP53* WT | *TP53* Mut |
| *ESR1* WT | 25 | 10 | | *ESR1* WT | 594 | 43 |
| *ESR1* Mut | 3 | 0 | | *ESR1* Mut | 161 | 6 |
| Soft Tissue Metastasis Only (n=26) | | | | *TP53* Other Missense (n=1,007) | | |
| | *TP53* WT | *TP53* Mut | | | *TP53* WT | *TP53* Mut |
| *ESR1* WT | 16 | 7 | | *ESR1* WT | 594 | 233 |
| *ESR1* Mut | 3 | 0 | | *ESR1* Mut | 161 | 19 |

Supplementary Table 1. Sample numbers separated by *ESR1* and *TP53* genotypes in different subset of tumors merged from six cohorts shown in Figure 1A.

**Supplementary Table 2**

| P53-ER Signature | |
|---|---|
| ABAT | MAST4 |
| ABCA3 | MED13L |
| ADCY1 | MLPH |
| AMFR | MPPED2 |
| ANXA9 | MUC1 |
| ARL3 | MYB |
| ASB13 | MYOF |
| BAG1 | NCOR2 |
| BCL2 | NPY1R |
| BHLHE40 | NRIP1 |
| CA12 | OLFM1 |
| CCND1 | OVOL2 |
| CD44 | PDZK1 |
| CELSR2 | PGR |
| CHPT1 | RAB17 |
| EGR3 | RARA |
| ELF1 | RBBP8 |
| ELOVL2 | REEP1 |
| ELOVL5 | RPS6KA2 |
| FARP1 | SEC14L2 |
| FLNB | SEMA3B |
| FOS | SLC22A5 |
| GFRA1 | SLC27A2 |
| GREB1 | SLC39A6 |
| IGF1R | SLC7A2 |
| IGFBP4 | SNX24 |
| IL17RB | STC2 |
| IL6ST | SYBU |
| ITPK1 | SYNGR1 |
| KCNK15 | TFF1 |
| KDM4B | TFF3 |
| KRT15 | THSD4 |
| LRIG1 | TJP3 |
| MAPT | UGCG |
| | WFS1 |
| | XBP1 |

| Non-P53-ER Signature | |
|---|---|
| ABHD2 | MED24 |
| ABLIM1 | MICB |
| AKAP1 | MREG |
| ALDH3B1 | MSMB |
| AQP3 | MYBBP1A |
| AREG | MYC |
| B4GALT1 | OLFML3 |
| BLVRB | PDLIM3 |
| CALB2 | PEX11A |
| CALCR | PTGES |
| CANT1 | RHOBTB3 |
| CBFA2T3 | SCARB1 |
| CLDN7 | SH3BP5 |
| CYP26B1 | SLC24A3 |
| DEPTOR | SLC26A2 |
| DHRS2 | SLC9A3R1 |
| DHRS3 | SOX3 |
| ENDOD1 | SULT2B1 |
| ESRP2 | SYT12 |
| FASN | TIAM1 |
| FKBP4 | TIPARP |
| FOXC1 | TOB1 |
| FRK | TUBB2B |
| GAB2 | UNC119 |
| HR | WWC1 |
| HSPB8 | |
| INHBB | |
| INPP5F | |
| ISG20L2 | |
| KAZN | |
| KCNK5 | |
| KLF10 | |
| KLK10 | |
| KRT13 | |
| KRT18 | |

Supplementary Table 2. Gene list of p53-associated E2 response genes and non-p53-associated E2 response genes derived in this study.

**Supplementary Table 3**

| Cell Line | *TP53* Status | Cell Line | *TP53* Status |
|---|---|---|---|
| 21MT1 | Mut | 600MPE | WT |
| 21MT2 | Mut | HCC712 | WT |
| 21NT | Mut | KPL-1 | WT |
| 21PT | Mut | LY2 | WT |
| BT-20 | Mut | MCF7 | WT |
| BT-474 | Mut | MDA-MB-175-VII | WT |
| BT-483 | Mut | MDA-MB-361 | WT |
| CAMA-1 | Mut | UACC-812 | WT |
| EFM-19 | Mut | ZR-75-1 | WT |
| EFM-192A | Mut | ZR-75-30 | WT |
| HCC1008 | Mut | ZR75B | WT |
| HCC1143 | Mut | | |
| HCC1419 | Mut | | |
| HCC1428 | Mut | | |
| HCC1500 | Mut | | |
| HCC1599 | Mut | | |
| HCC1806 | Mut | | |
| HCC1937 | Mut | | |
| HCC1954 | Mut | | |
| HCC70 | Mut | | |
| HDQ-P1 | Mut | | |
| JIMT-1 | Mut | | |
| MDA-MB-134-VI | Mut | | |
| MDA-MB-330 | Mut | | |
| MDA-MB-415 | Mut | | |
| SKBR5 | Mut | | |
| SUM229PE | Mut | | |
| SUM44 | Mut | | |
| SUM52PE | Mut | | |
| T-47D | Mut | | |
| UACC-893 | Mut | | |

Supplementary Table 3. List of breast cancer cell lines with the corresponding *TP53* genotypes used in this study.

**Supplementary Table 4**

| *ESR1*-targeting miRNA | | |
|---|---|---|
| hsa-mir-934 | hsa-mir-519d | hsa-mir-873 |
| hsa-mir-18a | hsa-mir-548j | hsa-mir-885 |
| hsa-mir-224 | hsa-mir-301b | hsa-mir-204 |
| hsa-mir-381 | hsa-mir-548f-1 | hsa-mir-548l |
| hsa-mir-299 | hsa-mir-1255a | hsa-mir-211 |
| hsa-mir-432 | hsa-mir-520c | hsa-mir-651 |
| hsa-mir-187 | hsa-mir-891a | hsa-mir-18b |
| hsa-mir-495 | hsa-mir-1468 | hsa-mir-3122 |
| hsa-mir-500b | hsa-mir-668 | hsa-mir-582 |
| hsa-mir-2115 | hsa-mir-548b | hsa-mir-520b |
| hsa-mir-376c | hsa-mir-520d | hsa-mir-573 |
| hsa-mir-17 | hsa-mir-4278 | hsa-mir-3618 |
| hsa-mir-656 | hsa-mir-3120 | hsa-mir-373 |
| hsa-mir-450a-1 | hsa-mir-526b | hsa-mir-570 |
| hsa-mir-106b | hsa-mir-519b | hsa-mir-337 |
| hsa-mir-20a | hsa-mir-520f | hsa-mir-302c |
| hsa-mir-3691 | hsa-mir-545 | hsa-mir-552 |
| hsa-mir-130a | hsa-mir-524 | hsa-mir-1909 |
| hsa-mir-2355 | hsa-mir-3616 | hsa-mir-3184 |
| hsa-mir-374a | hsa-mir-302b | hsa-mir-181d |
| hsa-mir-450b | hsa-mir-590 | hsa-mir-554 |
| hsa-mir-376b | hsa-mir-1827 | hsa-mir-4264 |
| hsa-mir-519a-2 | hsa-mir-130b | hsa-mir-215 |
| hsa-mir-942 | hsa-mir-188 | hsa-mir-3183 |
| hsa-mir-520a | hsa-mir-152 | hsa-mir-302a |
| hsa-mir-19a | hsa-mir-3145 | hsa-mir-1915 |
| hsa-mir-93 | hsa-mir-22 | hsa-mir-3646 |
| hsa-mir-221 | hsa-mir-548x | hsa-mir-539 |
| hsa-mir-4310 | hsa-mir-181c | hsa-mir-1227 |
| hsa-mir-1257 | hsa-mir-20b | |

Supplementary Table 4. miRNA targeting *ESR1* transcript and negatively correlated with *ESR1* mRNA in TCGA ER+ tumors derived in this study. Previously reported *ESR1* miRNAs are labelled in red.

**Supplementary Table 5**

| *TP53* Mut | LOS Score | LOS Type | *TP53* Mut | LOS Score | LOS Type | *TP53* Mut | LOS Score | LOS Type |
|---|---|---|---|---|---|---|---|---|
| Q165H | 91.21 | Weak | R273S | 12.48 | Weak | G245D | 4.22 | Medium |
| L137Q | 86.61 | Weak | M246L | 12.23 | Weak | G245S | 4.21 | Medium |
| S20L | 86.03 | Weak | I195T | 11.93 | Weak | V274G | 4.05 | Medium |
| K120R | 69.45 | Weak | V143G | 11.50 | Weak | E285K | 4.01 | Medium |
| S215fs | 60.12 | Weak | R280G | 11.50 | Weak | V274L | 3.94 | Medium |
| A347T | 54.38 | Weak | G262V | 11.45 | Weak | Y236C | 3.57 | Medium |
| W270S | 51.28 | Weak | L344P | 10.59 | Weak | L194P | 3.36 | Medium |
| R181H | 47.54 | Weak | A347P | 10.58 | Weak | Y220C | 2.76 | Medium |
| P151L | 44.95 | Weak | K132R | 10.32 | Weak | D281E | 2.56 | Medium |
| R174G | 40.33 | Weak | V157F | 10.12 | Weak | P278S | 2.46 | Medium |
| C176F | 37.82 | Weak | C176W | 10.09 | Weak | R273H | 2.43 | Medium |
| R196G | 33.86 | Weak | T253P | 9.94 | Medium | P278R | 1.79 | Medium |
| E180K | 33.70 | Weak | R342P | 9.72 | Medium | G266E | 1.66 | Medium |
| K139N | 32.50 | Weak | Y234C | 9.62 | Medium | G279E | 1.13 | Medium |
| S215T | 31.14 | Weak | H193R | 9.42 | Medium | P278T | 0.93 | Strong |
| G199E | 27.87 | Weak | C141Y | 9.30 | Medium | Y234H | 0.90 | Strong |
| H179Y | 27.06 | Weak | H193L | 9.15 | Medium | V173L | 0.89 | Strong |
| H179R | 25.23 | Weak | T125K | 9.11 | Medium | R273C | 0.68 | Strong |
| E271K | 23.45 | Weak | R175H | 9.10 | Medium | F270C | 0.57 | Strong |
| A161T | 22.97 | Weak | P151H | 9.01 | Medium | R280K | 0.56 | Strong |
| L130F | 18.76 | Weak | C242F | 8.95 | Medium | Y234N | 0.51 | Strong |
| M246I | 18.15 | Weak | Y163C | 8.72 | Medium | R213Q | 0.43 | Strong |
| F270L | 18.03 | Weak | T155N | 8.65 | Medium | R248Q | 0.22 | Strong |
| H168L | 17.20 | Weak | F134V | 8.61 | Medium | D281H | 0.20 | Strong |
| V272M | 17.11 | Weak | K132N | 8.55 | Medium | S241F | 0.14 | Strong |
| G244D | 16.72 | Weak | V157G | 8.47 | Medium | D281N | 0.13 | Strong |
| H193Y | 16.64 | Weak | C176R | 8.44 | Medium | R282W | 0.13 | Strong |
| R337C | 16.27 | Weak | C135Y | 8.32 | Medium | R273L | 0.11 | Strong |
| A159V | 16.06 | Weak | F113V | 8.31 | Medium | R248W | 0.09 | Strong |
| C141W | 16.04 | Weak | V173M | 8.13 | Medium | R280T | 0.08 | Strong |
| E286K | 15.66 | Weak | R110P | 8.08 | Medium | L265P | 0.06 | Strong |
| Y205F | 15.13 | Weak | V216L | 7.89 | Medium | R282P | 0.01 | Strong |
| C238Y | 14.91 | Weak | P151A | 7.86 | Medium | C242Y | 0.00 | Strong |
| N239D | 14.59 | Weak | R175G | 7.85 | Medium | G244V | 0.00 | Strong |
| T211P | 13.84 | Weak | D208V | 7.31 | Medium | G245V | 0.00 | Strong |
| V216E | 13.81 | Weak | M246V | 7.18 | Medium | | | |
| Y236H | 13.77 | Weak | V274A | 6.70 | Medium | | | |
| N239S | 13.60 | Weak | R248P | 6.10 | Medium | | | |
| F109C | 13.43 | Weak | E286V | 4.86 | Medium | | | |

Supplementary Table 5. *TP53* missense mutations with their loss-of-function score and loss-of-function type classification used in this study.

**Supplementary Table 6**

| *TP53* gain-of-function missense mutations | Other *TP53* missense mutations | | |
|---|---|---|---|
| R175H | A159V | G279E | R342P |
| R273H | A161T | H168L | S20L |
| R208K | A347P | H193R | S215T |
| R248W | A347T | H193Y | T125K |
| R249S | C141W | I195T | T155N |
| H193L | C141Y | K120R | T211P |
| L194F | C176F | K132N | T253P |
| R248Q | C176R | K132R | V143G |
| R282W | C176W | K139N | V157G |
| R273L | C238Y | L130F | V173L |
| R267P | C242F | L137Q | V173M |
| R273C | C242Y | L194P | V216E |
| D281G | D186N | L265P | V216L |
| R249S | D208V | L344P | V272M |
| D42Y | D281E | M246I | V274A |
| R337H | D281H | M246L | V274G |
| V143A | D281N | M246V | V274L |
| G245H | D42N | N239D | W270S |
| H179G | E180K | N239H | Y163C |
| M273I | E224D | N239S | Y205F |
| H179R | E271K | P151A | Y234C |
| R248L | E271Q | P151H | Y234H |
| R172H | E285K | P151L | Y234N |
| V157F | E285L | P278R | Y236C |
| G245C | E286K | P278S | Y236H |
| C227F | E286V | P278T | |
| G266E | E287D | Q165H | |
| C277F | E294L | R110P | |
| R172H | F109C | R174G | |
| H179Y | F113V | R175G | |
| Y220C | F134V | R181H | |
| I245R | F270C | R196G | |
| R175P | F270L | R213Q | |
| C135Y | G199E | R248P | |
| S241F | G244D | R273S | |
| P273H | G244V | R280G | |
| C242R | G245D | R280K | |
| T155P | G245S | R280T | |
| C238F | G245V | R282P | |
| C176S | G262V | R337C | |

Supplementary Table 6. A list of *TP53* missense mutations sub-grouped by gain-of-function mutation or other missense mutations.
