# Supplementary material for: Mutual exclusivity of ESR1 and TP53 mutations in endocrine resistant metastatic breast cancer
Source: NPJ Breast Cancer. 2022 May 10;8:62. doi: 10.1038/s41523-022-00426-w (PMC9090919; doi:10.1038/s41523-022-00426-w)
Supplement: Supplementary file 2 — Reporting Summary Checklist [file 41523_2022_426_MOESM2_ESM.pdf]

nature portfolio

Corresponding author(s): Steffi Oesterreich

Last updated by author(s): Mar 24, 2022

Reporting Summary

Nature Portfolio wishes to improve the reproducibility of the work that we publish. This form provides structure for consistency and transparency in reporting. For further information on Nature Portfolio policies, see our Editorial Policies and the Editorial Policy Checklist.

Statistics

For all statistical analyses, confirm that the following items are present in the figure legend, table legend, main text, or Methods section.

n/a	Confirmed	
☐	☒	The exact sample size (*n*) for each experimental group/condition, given as a discrete number and unit of measurement
☐	☒	A statement on whether measurements were taken from distinct samples or whether the same sample was measured repeatedly
☐	☒	The statistical test(s) used AND whether they are one- or two-sided<br>*Only common tests should be described solely by name; describe more complex techniques in the Methods section.*
☐	☒	A description of all covariates tested
☐	☒	A description of any assumptions or corrections, such as tests of normality and adjustment for multiple comparisons
☐	☒	A full description of the statistical parameters including central tendency (e.g. means) or other basic estimates (e.g. regression coefficient) AND variation (e.g. standard deviation) or associated estimates of uncertainty (e.g. confidence intervals)
☐	☒	For null hypothesis testing, the test statistic (e.g. *F*, *t*, *r*) with confidence intervals, effect sizes, degrees of freedom and *P* value noted<br>*Give P values as exact values whenever suitable.*
☒	☐	For Bayesian analysis, information on the choice of priors and Markov chain Monte Carlo settings
☒	☐	For hierarchical and complex designs, identification of the appropriate level for tests and full reporting of outcomes
☐	☒	Estimates of effect sizes (e.g. Cohen's *d*, Pearson's *r*), indicating how they were calculated

Our web collection on statistics for biologists contains articles on many of the points above.

Software and code

Policy information about availability of computer code

Data collection	Vectra 3 Automated Quantitative Pathology Imaging System was used for multicolor imaging collection and quantification.
Data analysis	Data were analyzed using commercially available software and packages:<br>Basic calculation and visualization: Microsoft Excel 2020; Graphpad Prism (Version 7); R (Version 3.6.1), ggpubr (Version 0.4.0)<br>Staining quantification: inForm (Version 2.3)<br>RNA-seq analysis: GSVA (Version 1.42.0), edgeR (Version 3.36.0), matrixTests (Version 0.1.9.1)<br>ChIP-seq analysis: DiffBind (Version 3.4.7), regionR (Version 1.26.1), BSgenome.Hsapiens.UCSC.hg38 (Version 1.4.4)

For manuscripts utilizing custom algorithms or software that are central to the research but not yet described in published literature, software must be made available to editors and reviewers. We strongly encourage code deposition in a community repository (e.g. GitHub). See the Nature Portfolio guidelines for submitting code & software for further information.

Data

Policy information about availability of data

All manuscripts must include a data availability statement. This statement should provide the following information, where applicable:

- Accession codes, unique identifiers, or web links for publicly available datasets
- A description of any restrictions on data availability
- For clinical datasets or third party data, please ensure that the statement adheres to our policy

All data analyzed in this study have previously been reported and are publicly available: ESR1 and TP53 mutation annotation results of MSKCC, INSERM, TCGA and METABRIC cohorts were directly downloaded from cBioPortal (https://www.cbioportal.org/) (Ref 101). Mutation data from POG570 (Ref 41) and MET500 (Ref 40) cohorts were obtained from the specific web-portals (https://met500.path.med.umich.edu) and (https://www.bcgsc.ca/downloads/POG570/), and mutation matrix

from METAMORPH and samples from the UNC Rapid Autopsy program were obtained from the original publications (Ref 42,65). "UC" is our in-house cohort and mutations were called as previously described (Ref 23). For the MET500 cohort (Ref 40), RNA-seq fastq files from 91 metastatic breast cancer samples were downloaded from the Database of Genotypes and Phenotypes (dbGaP) with accession number phs000673.v2.p1 (https://www.ncbi.nlm.nih.gov/projects/gap/cgi-bin/study.cgi?study_id=phs000673.v2.p1). For the POG570 cohort (Ref 41), raw count matrixes and mutation statuses were downloaded from the BCGSC portal. RNA-seq data and clinical information from TCGA and METABRIC were obtained from the GSE62944 and Synapse software platform under accession number syn1688369 (https://www.synapse.org/#!Synapse:syn1688369/wiki/27311) respectively. TCGA RPPA and miRNA-seq data were directly downloaded from FireBroswe (http://firebrowse.org/). For pan-breast cancer cell line transcriptomic clustering, 97 breast cancer cell line RNA-seq data were merged from three studies (Ref 92-94) . In vitro ER+ cell line TP53 knockdown microarray data were obtained from GSE3178 (Ref 52). Nutlin-treated MCF7 RNA-seq data sets were downloaded from GSE47042 (Ref 53) and GSE86221 (Ref 51). Original TP53 ChIP-seq data were released from GSE109482 (deposited but not published), GSE86164 (Ref 51), GSE100292 (Ref 60) and GSE47041(Ref 53). P53 ChIP-seq and GRO-seq with nutlin treatment were obtained from GSE86164 (Ref 51) and GSE53499 (Ref 61). ER ChIP-seq used for track visualization from MCF7 and ZR75-1 were obtained from GSE32222 (Ref 62). Other ER ChIP-seq data for intersection analysis were downloaded from GSE75779 (Ref 64) and GSE103023 (Ref 63).

Field-specific reporting

Please select the one below that is the best fit for your research. If you are not sure, read the appropriate sections before making your selection.

☒ Life sciences ☐ Behavioural & social sciences ☐ Ecological, evolutionary & environmental sciences

For a reference copy of the document with all sections, see nature.com/documents/nr-reporting-summary-flat.pdf

Life sciences study design

All studies must disclose on these points even when the disclosure is negative.

Sample size	No statistical test was used to predetermine sample size.
Data exclusions	No data were excluded in this study.
Replication	Wetbench experiments including multicolor staining, dual-color IF staining and p53 immunohistology were all done once due to the limited amount of clinical samples.
Randomization	No randomization was performed in this study.
Blinding	No blinding was performed in this study.

Reporting for specific materials, systems and methods

We require information from authors about some types of materials, experimental systems and methods used in many studies. Here, indicate whether each material, system or method listed is relevant to your study. If you are not sure if a list item applies to your research, read the appropriate section before selecting a response.

Materials & experimental systems

n/a	Involved in the study	
☐	☒	Antibodies
☒	☐	Eukaryotic cell lines
☒	☐	Palaeontology and archaeology
☒	☐	Animals and other organisms
☐	☒	Human research participants
☒	☐	Clinical data
☒	☐	Dual use research of concern

Methods

n/a	Involved in the study	
☒	☐	ChIP-seq
☒	☐	Flow cytometry
☒	☐	MRI-based neuroimaging

Antibodies

Antibodies used	p53 (Cell Marque, #453M-94, 1:500); CD4 (Agilent Cat# M7310, RRID:AB_2728838, 1.4 µg/ml), Foxp3 (Abcam Cat# ab20034, RRID:AB_445284, 1:200), CD8 (Agilent Cat# M7103, RRID:AB_2075537, 0.4 µg/ml), CD20 (Abcam Cat# ab9475, RRID:AB_307267, 1:300); CD68 (Agilent Cat# GA60961-2, RRID:AB_2661840, 0.12 µg/ml), and pan cytokeratin (Agilent Cat# M3515, RRID:AB_2132885, 0.18 µg/ml); PD-L1 (Abcam Cat# ab228462, RRID:AB_2827816,1:400).
Validation	Validation of all the commercial antibodies can be found on the manufacturer's website using the provided catalog number.

Human research participants

Policy information about studies involving human research participants

Population characteristics	1. ER+/HER2- measurable or evaluable metastatic breast cancer female patients without central nervous system disease<br>2. Enrolled in clinical trial NCT02953860<br>3. Median age 61 yeas (46-87)<br>4. A median of 2 prior chemotherapy and 2 prior hormonal therapies for metastatic disease
Recruitment	Interested participants were given a copy of the applicable consent for review and an IRB-approved flyer/brochure that summarizes the research procedures. Core needle biopsies were acquired from patients, who gave their informed written consent
Ethics oversight	COMIRB 16-1001

Note that full information on the approval of the study protocol must also be provided in the manuscript.
